# Multiple Quantitative Trait Loci Influence the Shape of a Male-Specific Genital Structure in *Drosophila melanogaster*

**DOI:** 10.1534/g3.111.000661

**Published:** 2011-10-01

**Authors:** Casey L. McNeil, Clint L. Bain, Stuart J. Macdonald

**Affiliations:** Department of Molecular Biosciences, University of Kansas, Lawrence, Kansas 66045

**Keywords:** QTL mapping, sexual selection, morphometric analysis, evolution

## Abstract

The observation that male genitalia diverge more rapidly than other morphological traits during evolution is taxonomically widespread and likely due to some form of sexual selection. One way to elucidate the evolutionary forces acting on these traits is to detail the genetic architecture of variation both within and between species, a program of research that is considerably more tractable in a model system. *Drosophila melanogaster* and its sibling species, *D. simulans*, *D. mauritiana*, and *D. sechellia*, are morphologically distinguishable only by the shape of the posterior lobe, a male-specific elaboration of the genital arch. We extend earlier studies identifying quantitative trait loci (QTL) responsible for lobe divergence across species and report the first genetic dissection of lobe shape variation within a species. Using an advanced intercross mapping design, we identify three autosomal QTL contributing to the difference in lobe shape between a pair of *D. melanogaster* inbred lines. The QTL each contribute 4.6–10.7% to shape variation, and two show a significant epistatic interaction. Interestingly, these intraspecific QTL map to the same locations as interspecific lobe QTL, implying some shared genetic control of the trait within and between species. As a first step toward a mechanistic understanding of natural lobe shape variation, we find an association between our QTL data and a set of genes that show sex-biased expression in the developing genital imaginal disc (the precursor of the adult genitalia). These genes are good candidates to harbor naturally segregating polymorphisms contributing to posterior lobe shape.

There is a great deal of interest in characterizing the morphological and behavioral changes that distinguish closely related species to understand the evolutionary processes involved in the early stages of speciation. In this context, the male genitalia of insects have come under particular scrutiny due to the observation that genital morphology is often species specific and can show striking diversity across related taxa that are otherwise similar in form (Eberhard 1985). Various lines of evidence point to sexual selection as a likely driver of this rapid divergence in genital morphology (Eberhard 1985, 2010; Hosken and Stockley 2004). Although the precise mechanism of sexual selection is debated, a popular hypothesis is cryptic female choice (Eberhard *et al.* 1998; Eberhard 2010). Indeed, several studies have demonstrated that variation in male fertilization success is linked to morphological variation in male genital structures (*e.g.*, Arnqvist and Danielsson 1999; Danielsson and Askenmo 1999; reviewed by Eberhard 2011). Despite interest in the variation and evolution of male genitalia, few studies have attempted to genetically dissect these traits (however, see Sasabe *et al.* 2010 and Schafer *et al.* 2011). Describing the genetic loci responsible for phenotypic variation in terms of their allelic effects, population frequencies, and interactions can provide valuable information about the evolutionary forces acting on a trait (Templeton 1981; Mitchell-Olds *et al.* 2007).

*Drosophila melanogaster* is one of a handful of elite model genetic systems, and it has been widely employed to characterize the genetic architecture of trait variation (Flint and Mackay 2009; Mackay 2010). Numerous related Drosophilid species can also be reared easily in the laboratory, and recent large-scale sequencing efforts have generated genome sequences for many of them (Clark *et al.* 2007), increasing their utility as experimental organisms for comparative work. In addition, the three species—*D. mauritiana*, *D. sechellia*, and *D. simulans*—most closely related to *D. melanogaster* are reproductively interfertile, allowing recombinant individuals to be produced for genetic analysis of traits distinguishing the species. Thus, this species group provides an excellent platform with which to understand the forces that shape phenotypic variation.

Interestingly, all four species of the *D. melanogaster* complex are morphologically very similar except for the shape and size of an elaborate cuticular projection (the posterior lobe) on the male genital arch, a structure that is the only reliable morphological indicator of species identity (Ashburner *et al.* 2005). The posterior lobe inserts under the ninth abdominal tergite of the female during copulation (Robertson 1988) and is used by the male during mounting and the early stages of mating to maintain strong genital coupling (Jagadeeshan and Singh 2006). Although no formal association has been made between posterior lobe morphology and male mating success, the striking variation across species suggests directional sexual selection acting on the structure. Thus, we have an opportunity to genetically dissect a rapidly evolving, male-specific genital trait using the armamentarium of genetic tools available for the *Drosophila* experimental system.

A number of studies have examined divergence between *Drosophila* species in posterior lobe morphology using quantitative trait locus (QTL) mapping techniques. Early work confirmed that interspecific variation for the trait is polygenic, with separate crosses between *D. simulans* and *D. melanogaster*, *D. mauritiana*, and *D. sechellia* all identifying at least one genetic factor contributing to phenotypic variation on each of the three major chromosomes (Coyne 1983; Coyne and Kreitman 1986). Subsequent work using larger panels of recombinants and genome-wide sets of markers identified multiple QTL on each chromosome for the *D. simulans* × *D. mauritiana* cross (Liu *et al.* 1996; Laurie *et al.* 1997; Zeng *et al.* 2000) and the *D. simulans* × *D. sechellia* cross (Macdonald and Goldstein 1999). Collectively, these studies suggest that QTL contributing to posterior lobe divergence between species are numerous, show limited epistasis, and are predominantly additive. Strikingly, additive effects were nearly always in the same direction: substituting a *D. simulans* allele for a *D. mauritiana* or a *D. sechellia* allele at a lobe QTL always gave a more *D. simulans*–like lobe phenotype. This suggests a consistent history of strong directional selection acting on the trait during species divergence (Orr 1998).

Despite the work on interspecific variation in genital morphology between members of the *D. melanogaster* complex, no study has yet described natural genetic variation for the posterior lobe within any one of these species. If we can characterize the loci that maintain the subtle lobe shape variation within a species, as well as those that influence extreme posterior lobe diversification among species, we can elucidate the relationship between intra- and interspecific genetic variation and develop a detailed understanding of the selective forces operating on the trait. In this study, we survey a series of *D. melanogaster* inbred lines and find considerable variation in posterior lobe morphology. We then carry out QTL mapping, employing an advanced generation recombinant population to genetically dissect variation between a pair of lines that differ in posterior lobe shape.

## Materials and Methods

### *D. melanogaster* stocks

Fifteen highly inbred, *P*-element and *Wolbachia*-free lines were used in this study. Fourteen were obtained from stock centers and were subjected to multiple generations of brother-sister mating prior to this study (see Table 1 of Macdonald and Long 2007). The remaining isogenic line, *Samarkand ry^506^* (hereafter, *Sam*), which harbors a mutant eye-color allele at the third chromosome *rosy* locus, was provided by T.F.C. Mackay and is described in Lyman *et al.* (1996).

**Table 1:** Posterior lobe morphology shows variation across *D. melanogaster* strains

Strain*^a^*	*N**^b^*	Lobe area (× 10^−3^ mm^2^)	Lobe height (× 10^−3^ mm)	Lobe width (× 10^−3^ mm)	H:W*^c^*	sPC1 (× 10^−4^)*^d^*	sPC2 (× 10^−4^)*^d^*	sPC3 (× 10^−4^)*^d^*
b1	24	3.43 (0.228)	56.0 (2.86)	59.0 (1.92)	0.95 (0.044)	37.2 (13.73)	−26.8 (15.15)	23.7 (17.29)
b3839	32	3.62 (0.174)	56.9 (2.22)	61.4 (1.92)	0.93 (0.046)	−7.7 (16.01)	−15.2 (15.06)	−4.5 (17.04)
b3841	35	3.32 (0.235)	51.7 (2.56)	62.4 (2.77)	0.83 (0.051)	−3.4 (20.82)	8.5 (13.00)	−6.6 (18.08)
b3844	36	3.17 (0.168)	51.1 (2.01)	60.5 (1.91)	0.84 (0.039)	16.3 (14.44)	13.5 (12.62)	10.7 (14.03)
b3846	25	3.67 (0.160)	51.5 (1.46)	70.0 (2.83)	0.74 (0.040)	31.9 (20.43)	8.7 (12.63)	−31.8 (13.41)
b3852	14	3.14 (0.153)	55.7 (2.40)	51.8 (1.54)	1.08 (0.046)	−94.3 (12.15)	12.1 (10.74)	−1.3 (14.89)
b3864	15	3.95 (0.170)	57.4 (2.20)	66.3 (2.26)	0.87 (0.051)	23.9 (16.62)	−33.8 (15.90)	−25.7 (15.10)
b3870	23	3.22 (0.155)	51.5 (1.81)	62.8 (1.56)	0.82 (0.029)	−7.9 (13.21)	22.0 (10.98)	1.7 (12.32)
b3875	14	3.64 (0.197)	58.1 (2.10)	62.2 (2.30)	0.94 (0.042)	−5.9 (15.99)	−14.1 (12.11)	−8.0 (12.36)
b3886	18	3.47 (0.242)	54.2 (2.94)	63.0 (2.23)	0.86 (0.054)	−14.1 (17.84)	8.6 (16.55)	−19.8 (13.97)
*Samarkand ry^506^*	13	3.06 (0.247)	46.0 (1.95)	66.2 (3.55)	0.70 (0.033)	62.8 (16.32)	34.9 (11.74)	18.1 (18.37)
t14021-0231.0	18	3.53 (0.194)	58.3 (2.34)	57.9 (2.30)	1.01 (0.050)	3.1 (18.63)	−26.2 (14.96)	8.4 (12.83)
t14021-0231.1	12	3.25 (0.126)	50.3 (1.83)	62.7 (1.42)	0.80 (0.035)	11.6 (11.63)	18.9 (10.66)	−4.8 (11.25)
t14021-0231.4	21	3.29 (0.250)	50.4 (2.87)	65.6 (2.35)	0.77 (0.045)	34.4 (24.00)	6.8 (11.92)	9.8 (19.07)
t14021-0231.7	42	3.21 (0.222)	54.7 (2.86)	55.0 (1.78)	1.00 (0.049)	−51.6 (16.87)	−8.2 (20.73)	12.2 (12.67)

Values represent the strain mean (standard deviation) for various measures of posterior lobe size and shape.

a Names of the 15 strains used. b3852 and *Samarkand ry^506^* were used as parents for the F_2_ QTL mapping study.

b Number of individuals phenotyped per strain. For each fly, the phenotype data from one lobe was used.

c H:W is the ratio of lobe height to lobe width.

d First three principal components from the species diversity PCA employing 100 elliptic Fourier coefficients per lobe. These represent orthogonal aspects of posterior lobe shape and explain 53.0% (sPC1), 18.8% (sPC2), and 16.3% (sPC3) of shape variation across the 15 strains. All other PCs explain less than 5% of lobe variation.

Unless otherwise stated, all flies were reared at 23° under constant light, using 10 ml of cornmeal-molasses-yeast medium in polystyrene vials (25 × 95 mm).

### Experimental flies

#### Survey of intraspecific variation in genital morphology:

For each of the 15 strains, we generated three or four replicate vials, collected males under CO_2_ anesthesia, and stored them at −20° in 1.5 ml microcentrifuge tubes until dissection. An average of 22.8 males were successfully phenotyped per strain (range = 12–42), with a mean of 6.84 per replicate vial.

#### F_2_ coarse-mapping population:

We chose b3852 and *Sam*, a pair of strains with divergent lobe morphology, and initiated multiple replicate cross-vials with 10 virgin b3852 females and 10 *Sam* males. Parental flies were removed within 48 hr to maintain a relatively constant low larval density. F_1_ hybrid progeny were collected and aged in single-sex groups to ensure females were virgin, and then multiple replicate intercross vials holding 10 virgin F_1_ females and 10 F_1_ males were set up. Again, flies were removed within 48 hr. Upon maturation, F_2_ males from each replicate vial were collected and frozen as described above.

#### F_17_ fine-mapping population:

Reciprocal crosses between b3852 and *Sam* were carried out in small polypropylene bottles (8 oz, 60 × 130 mm). Approximately 200 F_1_ individuals from each reciprocal cross were mixed, and the combined population was split into two fresh bottles. In the next generation, F_2_ flies were combined into a single large glass bottle (64 oz), and this recombinant population was maintained at high census size with 12–13 day generations until the F_16_ generation eclosed. A large number of replicate vials were each initiated with ∼20 F_16_ individuals, and flies were allowed to lay eggs for 24 hr. F_17_ males were collected and frozen as described.

### Phenotype acquisition

The terminalia was dissected from each experimental male, individually placed in a 0.2 ml PCR tube containing a drop of 1M KOH, and boiled for 2–5 min to dissolve unwanted connective tissue. For recombinant F_2_ and F_17_ flies, the remainder of the dissected animal was refrozen for subsequent DNA extraction. The genital arch, including the paired posterior lobes and lateral plates, was then dissected out in 1M KOH and mounted on a microscope slide under a coverslip in a small drop of Aqua-Mount (Lerner #13800 via VWR #41799-008). Slides were left overnight at 40–45° on a slide warmer with a ∼4 g weight pushing the coverslip down, and the next day a TIFF image of each slide-mounted posterior lobe was captured at 400× total magnification. The dissection of an experimental individual was considered successful if at least one of the pair of posterior lobes was undamaged.

For lobe data to be comparable across genotypes, lobes must be placed in a standard configuration prior to morphometric analysis; *i.e.*, all lobes should have the same handedness, orientation, and relative location. To ensure all lobes were of the same handedness, images were manually flipped such that the lateral plate (and thus the “point” of the lobe) points clockwise (refer to Figure 3). Each image was then manually outlined in *ImageJ* (Rasband 1997–2011) using a custom macro to automatically record a set of Cartesian coordinates defining each outline. Following previous work on the posterior lobe (Liu *et al.* 1996; Macdonald and Goldstein 1999; Masly *et al.* 2011), outlines were closed with an artificial baseline that extends from the point at which the lateral plate connects to the posterior lobe. Outlines from all lobes were subsequently oriented to make these baselines horizontal. Finally, the origin of each set of coordinates was placed at the centroid of the outline to make the locations of all lobe outlines comparable.

Due to the lack of reliable morphological landmarks on the posterior lobe, we used elliptic Fourier analysis (EFA) to describe outline shape (Kuhl and Giardina 1982; Ferson *et al.* 1985). We applied EFA using a custom *R* script (http://www.r-project.org/; R Development Core Team 2010). A detailed description of the methodology as applied to posterior lobe shape is provided in Liu *et al.* (1996) and Macdonald and Goldstein (1999). Briefly, elliptic Fourier functions use a parametric representation of the *x*- and *y*-projections of the outline, treating each independently as a function of contour length. Following EFA, each outline is represented by a set of 4*n* Fourier coefficients that can reproduce the outline with arbitrary precision depending on the number of harmonics (*n*). Here we use 25 harmonics, which provides a near-perfect reconstruction of the original outline (see Figure 2 in Liu *et al.* 1996) and yields 100 coefficients per lobe. Because we had placed the outlines in a standard configuration prior to EFA, in our analyses we did not employ the coefficient normalizing functions described in Kuhl and Giardina (1982). However, we obtained practically identical QTL mapping results for the mPC1 shape measure whether or not we applied these functions (data not shown). In addition, because posterior lobe morphology is largely unaffected by variation in overall body size (Liu *et al.* 1996; Macdonald and Goldstein 1999; Shingleton *et al.* 2009; Masly *et al.* 2011), we did not seek to control for such variation, for instance, by measuring wing area or tibia length.

The 100 Fourier coefficients for a subset of experimental individuals were treated as variables in a principal components analysis (PCA) to encapsulate shape variation in a small number of mathematical descriptors. Two separate PCA were carried out using the ‘prcomp’ *R* function, one for the species diversity experiment, and one for the mapping experiment. The species diversity PCA consisted of individuals from the set of 15 strains used to examine morphological variation within *D. melanogaster*. The mapping experiment PCA employed all mapping population flies and their progenitors (b3852, *Sam*, F_1_, F_2_, and F_17_). We caution that the principal component (PC) shape descriptors may not be comparable across these two analyses; to avoid confusion, we prefixed principal components derived from the species diversity PCA with an “s” (*e.g.*, sPC1) and those from the mapping experiment PCA with an “m” (*e.g.*, mPC1).

Finally, we estimated the size of each lobe as the area enclosed by the outline, lobe height (width) as the length of the vertical (horizontal) line through the centroid, and the height:width ratio (H:W) as the ratio of these two distances.

### Genetic markers

Markers discriminating b3852 and *Sam* were identified by sequencing a series of 1 kb PCR fragments in both lines. SNPs were submitted to the Illumina GoldenGate assay design tool, and 96 high-scoring SNPs spread along the three major chromosomes were chosen for genotyping in our F_2_ and F_17_ mapping panels (File S1). DNA was extracted from each phenotyped recombinant using the Puregene cell and tissue kit (Qiagen), resuspended in 20 µl of 1X TE, and 10 µl of diluted DNA was used for genotyping (Illumina BeadXpress platform, UC Davis Genome Center). The resulting raw intensity data were submitted to a custom set of *R* scripts to call genotypes (see Macdonald *et al.* 2005), and 87/96 SNPs yielded high-quality genotypes (X = 16, 2L = 22, 2R = 17, 3L = 12, 3R = 20). We also genotyped a single RFLP marker at the *eyeless* gene on chromosome 4 in the F_2_ mapping panel. Briefly, we amplified a short PCR fragment containing a diagnostic SNP (eyeF, 5′-TGT GTG AGC AAA ATT CTC GG-3′; eyeR, 5′-GTT TCG GCA TGG TAG GAC AT-3′), digested with *Mbo*II, and genotyped by separating restriction fragments on a 2.5% agarose gel.

### QTL mapping

For the recombinant flies, depending on the quality of the dissected material, phenotypes were scored on either one or both of the posterior lobes. When both lobes were successfully imaged (153/711 or 21.5% of the recombinants) we randomly chose the phenotype from a single lobe for mapping. All QTL mapping analyses and estimation of the genetic map from the marker genotypes were carried out within *R/qtl* (Broman and Sen 2009). Input files are available (see supporting information, File S2 and File S3). For the F_2_, both interval mapping (IM; Lander and Botstein 1989) and composite interval mapping (CIM; Zeng 1994) were performed using the multiple imputation method of Sen and Churchill (2001) with 256 imputations. Statistical significance was determined from 1000 permutations (Churchill and Doerge 1994), taking care to generate *X*- and autosome-specific thresholds (Broman *et al.* 2006). For the F_17_ we took a selective genotyping approach to fine-map QTL influencing the mPC1 measure of shape. Of the 344 phenotyped F_17_ males, the 47 with the lowest, most b3852-like mPC1 score, and the 47 with the highest, most *Sam*-like mPC1 score were genotyped. To minimize analytical bias associated with selective genotyping, all phenotyped F_17_ individuals were included in the QTL mapping analysis (IM using multiple imputation), with the genotypes from the nontail individuals recorded as missing (Lander and Botstein 1989; Sen *et al.* 2005). In addition, a stratified permutation test was carried out, separately permuting phenotypes within the genotyped and ungenotyped subsets of F_17_ individuals (Manichaikul *et al.* 2007).

## Results and Discussion

### Variation in posterior lobe morphology within *D. melanogaster*

The shape and size of the posterior lobe differ among the four members of the *melanogaster* complex of species (see Figure 1 in Liu *et al.* 1996). In addition to this dramatic interspecific variation, more subtle intraspecific variation has been noted for *D. simulans*, *D. mauritiana*, and *D. sechellia* (Liu *et al.* 1996; Macdonald and Goldstein 1999). We extend these surveys of variation to *D. melanogaster* and score individuals from 15 inbred lines to generate a framework for understanding the basis of lobe shape and size variation in this model genetic system.

**Figure 1:**
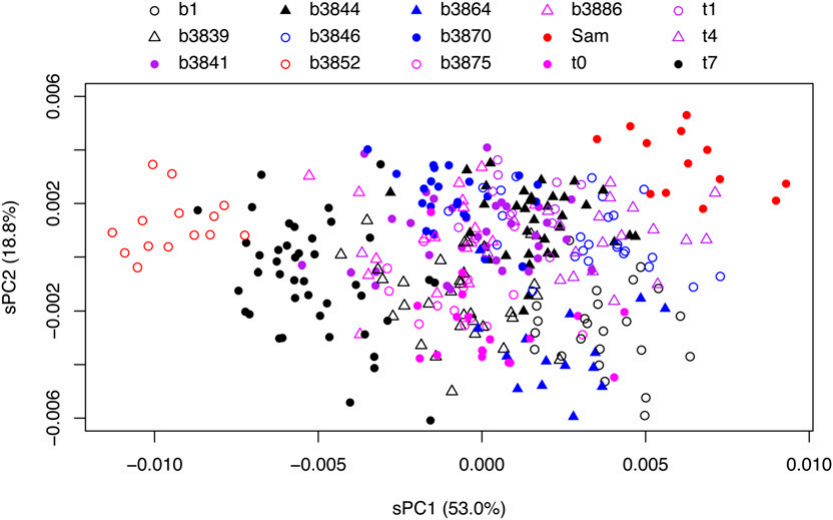
Variation in posterior lobe shape in *D. melanogaster*. Lobe outlines from a number of males (one lobe per individual) from 15 inbred lines were subjected to elliptic Fourier analysis, and the resulting coefficients used in a principal components analysis (the species diversity PCA). Considerable variation in shape among strains is shown for the two major principal components, sPC1 and sPC2. Strains in red symbols are those chosen as the parents for QTL mapping.

Because the posterior lobe lacks clear morphological landmarks, we quantified size and shape variation using morphometric analyses based on sets of Cartesian coordinates defining lobe outlines. An EFA of each outline results in a series of Fourier coefficients, and a PCA of these values encapsulates variation across individuals in a series of orthogonal descriptors of shape. Figure 1 highlights shape variation along two of these descriptors, sPC1 and sPC2, that together explain >70% of lobe variation in our sample of lines. While PCA provides a convenient small set of mathematical descriptors of shape, their interpretation is difficult due to the sheer complexity of the shape variation across lines (see Figure S1). Nonetheless, careful examination of the point clouds from Figure 1, along with the relevant columns from Table 1, shows clustering of individuals from the same line and clear differences among lines. For instance, lines t7 and b3846 are separated along the sPC1 axis, whereas lines b3870 and t0 are separated along the sPC2 axis. These results show that our morphometric descriptions of shape are robust and allow discrimination of the different lobe shapes found in various genotypes of *D. melanogaster*.

The lines chosen for our survey were collected from sites in 10 different countries and, hence, captured a large swath of the cosmopolitan genetic variation in *D. melanogaster*. However, because we did not sample multiple genotypes from the same population, we cannot assess relative levels of within- and between-population variation in the posterior lobe. It may be that the extent of posterior lobe variation we describe, perhaps due to some degree of local adaptation, is greater than would be observed within a single population. A more extensive survey of morphological variation, including multiple genotypes from multiple different populations, is needed to address this question.

A primary goal of our survey was to identify a pair of morphologically distinct lines that differ along a major axis of intraspecific phenotypic variation for use as the parents for a QTL mapping study. We selected lines b3852 and *Sam* for this purpose (red symbols in Figure 1). These lines have similar lobe areas, differ strongly in sPC1 (the major axis of shape variation in the diversity panel) but not in sPC2 or sPC3, with b3852 having taller and narrower posterior lobes than *Sam* (Table 1).

### Phenotypic description of mapping population genotypes

**Figure 2:**
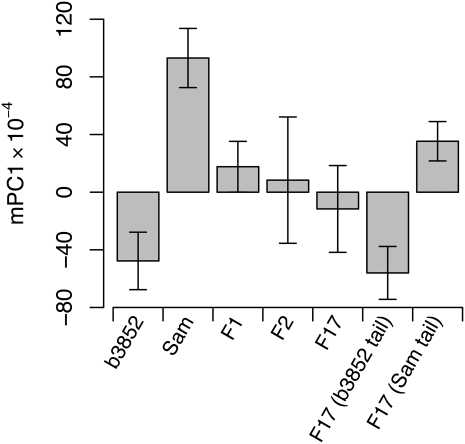
mPC1 lobe shape variation in b3852, *Sam*, F_1_, and recombinant genotype classes. Each bar shows the mean (± SD) of multiple individuals, taking just a single lobe from each fly: b3852 (*N* = 29), *Sam* (*N* = 25), F_1_ (*N* = 21), F_2_ (*N* = 367), F_17_ (*N* = 344), F_17_ b3852 tail (*N* = 47), and F_17_ Sam tail (*N* = 47). F_1_ males derived from reciprocal parental crosses have similar shapes and were averaged. The groups of F_17_ “tail” flies are the extreme individuals from either tail of the F_17_ phenotypic distribution.

Lines b3852 and *Sam* were intercrossed in separate experiments to generate F_2_ and F_17_ males. Posterior lobe outlines from all relevant genotypes (b3852, *Sam*, F_1_, F_2_, and F_17_) were processed via EFA, and the coefficients were used as variables in a PCA. The top six principal components each explain >1% of the posterior lobe variation among this set of individuals: mPC1 (62.6%), mPC2 (17.1%), mPC3 (12.2%), mPC4 (2.1%), mPC5 (1.8%), and mPC6 (1.2%). In addition, the parental strains are significantly different for each of the first four principal components: mPC1 (*t*-test, *P* < 1 × 10^−29^), mPC2, (*P* < 1 × 10^−4^), mPC3 (*P* = 0.003), and mPC4 (*P* < 1 × 10^−7^). However, the phenotypic distributions of the parental strains fail to overlap only for mPC1 (Figure 2).

**Figure 3:**
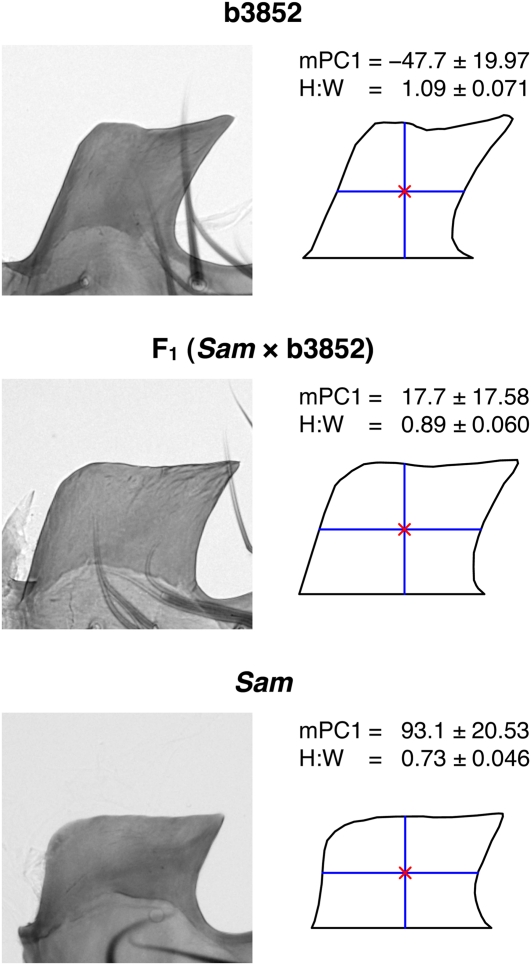
Morphology of the posterior lobe in the progenitors of the QTL mapping panels. On the left, a representative lobe image is presented for the two parental strains and the F_1_ hybrid (the result of a *Sam* female × b3852 male cross). The closed outlines derived from these images used for shape/size analysis are presented on the right. The red cross within each outline is the centroid, and blue lines represent lobe height and width. The mean (± SD) of the mPC1 (× 10^−4^) and height:width ratio (H:W) shape measures for each genotype highlight the inverse correlation between these two measures. Phenotype means are calculated from a single lobe from 29 b3852, 21 F_1_, and 25 *Sam* males.

mPC1 alone appears to provide the clearest descriptor of posterior lobe morphological variation in the b3852 × *Sam* cross. This finding is highlighted in Figure S2, which sorts the mapping population individuals by their mPC1 score and demonstrates a clear morphological transition from the b3852 lobe phenotype to the *Sam* lobe phenotype as mPC1 score increases. Because the correlation between lobe area and mPC1 is low (Table 2), we are able to consider lobe size and lobe shape (as measured by mPC1) as separate sources of morphological variation in this cross. Figure 2 shows the average mPC1 score in both parental lines, the F_1_, and both the F_2_ and F_17_ recombinant populations. As expected, the genetically variable samples show greater variation than the parentals and hybrids. In addition, the F_1_ hybrid males have a mPC1 phenotype that is midway between the parental line means, suggesting the trait is largely additive (see also Figure 3).

**Table 2:** Correlations among lobe phenotypes in the two mapping panels

	Area	Height	Width	H:W*^a^*	mPC1*^b^*	mPC2*^b^*	mPC3*^b^*
Area	—	0.63***	0.49***	−0.04	0.17	0.05	0.95***
Height	0.76***	—	−0.31	0.73***	−0.57**	0.26	0.70***
Width	0.56***	−0.04	—	−0.87***	0.81***	−0.39***	0.33***
H:W*^a^*	0.12	0.71***	−0.73***	—	−0.86***	0.42***	0.10
mPC1*^b^*	0.16	−0.35**	0.61***	−0.67***	—	NA	NA
mPC2*^b^*	0.04	0.20	−0.42***	0.43***	NA	—	NA
mPC3*^b^*	0.95***	0.70***	0.53***	0.10	NA	NA	—

Correlations between traits in the F_2_ are above the diagonal (*N* = 367), and correlations in the F_17_ are below the diagonal (*N* = 344). Only a single lobe was used from each individual. Asterisks are used to represent significance level (* = 1 × 10^−5^, ** = 1 × 10^−10^, *** = 1 × 10^−15^).

a H:W is the ratio between lobe height and lobe width.

b The three major principal components explain 62.6% (mPC1), 17.1% (mPC2), and 12.2% (mPC3) of the shape variation in the mapping experiment PCA. Principal components are orthogonal, so correlations among them using the full dataset will be zero by design (and NA values are presented). Although this is not true when considering only a subset of the individuals used in a PCA, there are no significant correlations among principal components in either the F_2_ or the F_17_ (data not shown).

Principal components can be difficult to interpret in terms of familiar shape concepts, and we sought to define what aspect of lobe shape mPC1 describes in this cross. We measured the height and width of each lobe as the vertical and horizontal distance through the outline centroid, respectively, and took the ratio of height:width (H:W). Figure 3 shows that H:W and mPC1 show a strong negative relationship in the parental lines and the F_1_, and we found a strong negative correlation between the traits in both the F_2_ and the F_17_ (*r* = −0.86 and −0.67, respectively; Table 2). Thus, this quite crude H:W shape measure describes much of the same shape variation encapsulated by mPC1 and allows us to think of mPC1 as predominantly describing how squat or slender a lobe is.

### Coarse QTL mapping

**Figure 4:**
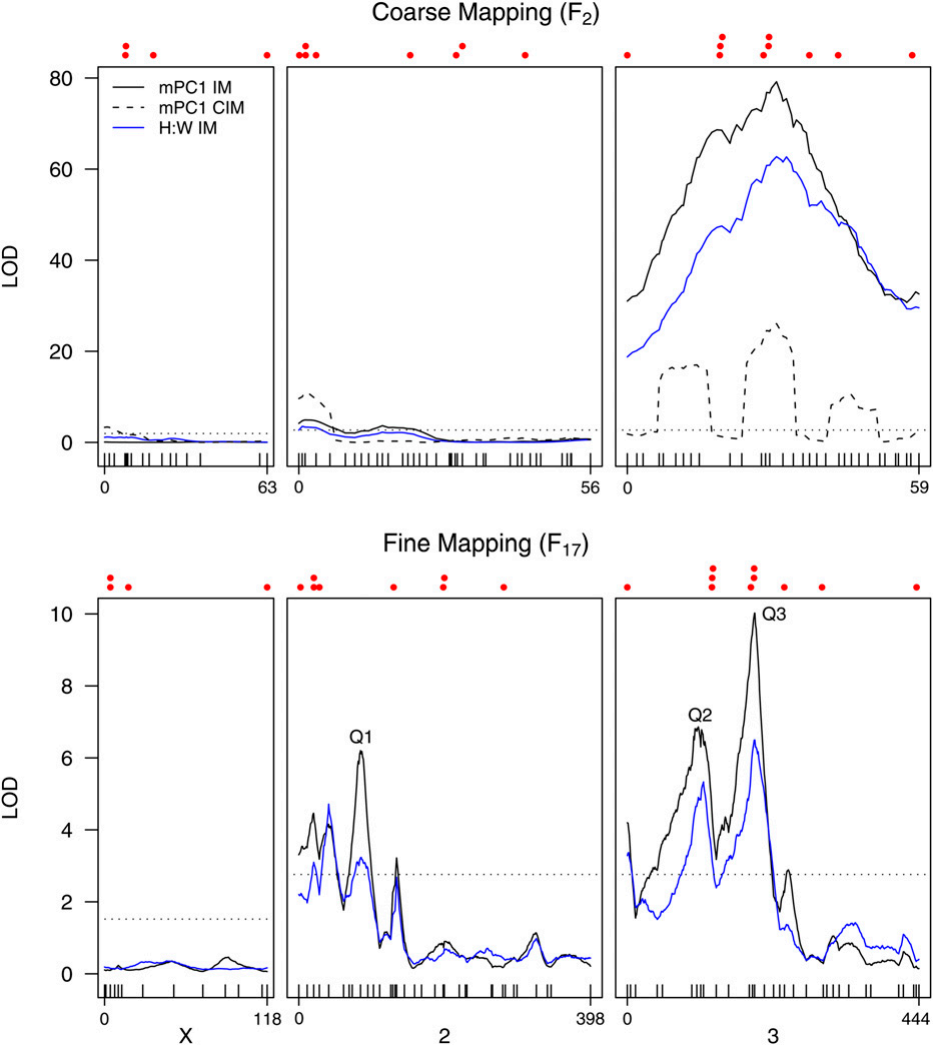
Autosomal QTL contribute to lobe shape variation between b3852 and *Sam*. Likelihood profiles from interval mapping (IM) are shown for mPC1 and the height:width ratio (H:W) for both the coarse- and fine-mapping experiments. In addition, the profile from composite interval mapping (CIM) is shown for mPC1 in the coarse-mapping experiment. The three major fine-mapped mPC1 QTL discussed in the text (Q1, Q2, Q3) are highlighted. The horizontal dotted line represents a 5% significance threshold, and as thresholds for each trait were very similar, we conservatively present only the highest threshold. The same set of 87 SNP markers was used for both mapping experiments (ticks along the X-axis), but care should be taken when comparing the two sets of plots as map lengths differ (given in cM, F_17_ length > F_2_ length), and relative marker spacing is not necessarily preserved. Above the likelihood profiles, we mark with solid red circles the positions of 22 plausible candidate genes that show sex-biased gene expression in genital discs (Chatterjee *et al.* 2011). These are (from left to right): X chromosome = *CG4766*, *Nep1*, *lz*, and *FucTC*; chromosome 2 = *al*, *CG4267*, *CG31686*, *msl-2*, *salr*, *ap*, *Wnt2*, and *Poxn*; chromosome 3 = *bab1*, *toe*, *eyg*, *caup*, *AP-2*, *dsx*, *Ctr1B*, *abd-A*, *bnl*, and *Dr*.

We first carried out standard F_2_ QTL mapping to provide a coarse map of loci contributing to morphological variation between b3852 (tall, narrow lobe) and *Sam* (low, broad lobe). Using interval mapping on 367 F_2_ individuals genotyped for a genome wide panel of markers, we identified an extremely strong QTL on chromosome 3 for mPC1 (LOD = 79.2 close to the centromere; top panel of Figure 4) and two smaller QTL near the tip of 2L (LOD = 4.9 and 3.7). These same QTL were also identified for the H:W shape measure, consistent with the strong correlation between this trait and mPC1 (Table 2). These QTL mapping analyses were conducted using the phenotypic score from only a single lobe per individual, but when we repeated the analysis and substituted data from the other lobe (if available), we identified the same QTL (Figure S3). This result was anticipated as there is a strong correlation between the mPC1 shape score for the paired lobes (*r* = 0.85).

Although IM has high power to identify QTL, it can provide unreliable estimates of the number and location of QTL (Zeng 1994; Cornforth and Long 2003). Therefore, we applied CIM to the mPC1 dataset to increase precision and further resolve QTL. Using a window size of 10 cM and four marker covariates, we found a novel QTL on the X, a single QTL at the tip of 2L, and resolved the broad third chromosome QTL into three separate QTL (Figure 4). However, by manipulating the window size and altering the number of markers fitted to the model, we found we could generate quite different LOD profiles, although all runs did include a large QTL interval spanning the chromosome 3 centromere.

IM was applied to all other phenotypes measured in the F_2_ (*i.e.*, lobe area, height, width, and mPC2-mPC6), regardless of the proportion of morphological variation explained or whether the trait discriminated the parental lines. The likelihood profiles shown in Figure S3 reveal a number of additional QTL underlying various aspects of lobe morphology. Notably, the lobe height and width LOD profiles are similar to those for mPC1 and H:W, reflecting the strong correlation between these traits (Table 2). The profiles for lobe area and mPC3 also follow each other closely, with QTL at the tip of the X and the middle of 3R, again due to a high positive correlation between the traits (Table 2).

Finally, we note that chromosome 4 failed to show a significant association with any trait tested in the F_2_ panel (data not shown).

### Fine-mapping mPC1 QTL

Ultimately, rather than applying additional statistical analyses to a standard F_2_ dataset, the best way to improve QTL mapping resolution, generate accurate estimates of QTL effects, and promote the identification of the causative nucleotide polymorphisms, is to increase the number of crossover events in the mapping population (*e.g.*, Cheng *et al.* 2010). Following Darvasi and Soller (1995), we generated an F_17_ advanced intercross line (AIL) between b3852 and *Sam*, passing the population through additional rounds of recombination (limited to females in *Drosophila*) to expand the genetic map by over 7-fold. We also elected to utilize a selective genotyping approach for the F_17_ population to reduce genotyping costs while maintaining high mapping power (Lander and Botstein 1989; Darvasi and Soller 1992). Because our goal was to fine-map QTL for mPC1, we chose to genotype subsets of the F_17_ individuals with mPC1 values most similar to the parental strains (F_17_ “tail” individuals in Figure 3). These individuals were genotyped for the same 87 SNP markers used for the F_2_, with all adjacent markers along a chromosome remaining linked on the expanded F_17_ genetic map.

Figure 4 (bottom panel) presents the results of fine mapping with IM for both mPC1 and the correlated H:W trait, showing similar results to the F_2_ map. The large pericentromeric chromosome 3 mPC1 QTL is preserved on fine mapping (Q3; LOD = 10.0); a second QTL on 3L (Q2; LOD = 6.8) is present in approximately the same location as the F_2_ CIM QTL; and there are QTL on chromosome 2L, including a relatively large QTL in the middle of 2L (Q1; LOD = 6.2). We found no evidence for a QTL in the middle of 3R in the F_17_ IM analysis similar to that we identified with CIM in the F_2_, either due to low power to detect it in the F_17_ or because the F_2_ CIM QTL was an artifact (we did not routinely map this QTL when varying the analysis parameters for CIM).

In considering fine-mapping power, it should be noted that during laboratory maintenance of the F_17_ population, either drift or selection led to a reduction in *Sam* allele frequency at various points along the genome. One indication of the skewed allele frequency is that the most *Sam*-like F_17_ flies are not as phenotypically extreme as the inbred *Sam* parent (Figure 2), implying a dearth of individuals homozygous for *Sam* alleles at loci contributing to posterior lobe variation. In addition, the frequency of *Sam* alleles is very low along the entire X chromosome and at the very telomeric end of 3R in all genotyped F_17_ flies (Figure S4), limiting our power to detect QTL in these regions. We hypothesize that the *Sam* genome carries slightly deleterious alleles at several loci and that individuals homozygous for these alleles were at a competitive disadvantage during creation of the AIL, resulting in a reduction in *Sam* allele frequency. Various multigenerational crossing designs can be used to create AILs while limiting the effects of drift and selection (*e.g.*, Rockman and Kruglyak 2008). Although more cumbersome than typical methods of maintaining large fly populations, such strategies are likely to be beneficial in maintaining a consistent level of mapping power along the genome in AIL-based QTL studies.

To explore our mPC1 QTL data further, we used various routines from *R/qtl* (Broman and Sen 2009), beginning with a simple model including the three QTL (Q1, Q2, and Q3) that each have LOD scores > 6 and in which we have the greatest confidence (Figure 4). The ‘addqtl’ function did not indicate that more QTL should be added to the model, although there was some suggestion of an additional QTL at the very tip of 2L. Using a combination of the ‘addint’ function, which asks whether allowing QTL to interact improves the model fit, and a direct two-dimensional scan for epistatic QTL with ‘scantwo,’ we found that Q1 and Q2 interact. The final model, *y* = Q1 + Q2 + Q3 + Q1 × Q2, explains an estimated 26.5% of the phenotypic variance (using the ‘fitqtl’ function). Each QTL contributes 4.6–10.7% to mPC1 variation (Table 3), and the Q1 × Q2 interaction contributes 5.5%. For all three QTL, substitution of a b3852 allele for a *Sam* allele increases mPC1 (giving a more *Sam*-like phenotype), with Q2 and Q3 acting predominantly additively and Q1 having a large dominance component (Table 3). It seems clear that no single QTL explains a large fraction of the morphological variation between the parental strains and that, instead, trait variation is conferred by the action of a number of relatively small-effect QTL. This is particularly true in light of our somewhat low F_17_ sample size, which has likely resulted in our overestimating QTL effects (Beavis 1994).

**Table 3:** Details of the fine-mapped mPC1 shape QTL

	Q1	Q2	Q3
Chromosome	2L	3L	3c*^a^*
Peak LOD	6.2	6.8	10.0
Variance explained (%)	10.7	8.2	4.6
Additive effect (× 10^−4^)	4.51	6.02	9.61
Dominance effect (× 10^−4^)	6.76	1.07	1.27
Interval (cM, expanded scale)	75–94	78–128	186–200
Interval (cM, regular scale)	25–31	22–38	45–50
Cytology	27E–29A	66B–69B	75F–86C
Physical size (Mb)	1.17	4.19	11.95
Number of genes	147 (7)	555 (19)	1,383 (69)

QTL intervals are based on a 2-LOD drop from each peak on the expanded F_17_ genetic map. The genetic intervals on the regular, unexpanded genetic map, as well as the cytological intervals, were inferred from the expanded genetic map by using the known positions of markers and physical-to-genetic distance conversion tables on FlyBase (Tweedie *et al.* 2009). The physical size of each QTL interval and the number of protein-coding genes (noncoding RNA genes) are also given.

a Implicated QTL interval spans the chromosome 3 centromere.

The goal of fine mapping is to reduce QTL map intervals, thereby promoting identification of the causative gene or polymorphism. We succeeded in expanding the map length of the autosomal genome by a factor of >7 between the F_2_ and F_17_ generations, and the confidence intervals for fine-mapped QTL are smaller than in the coarse-mapping study. Nonetheless, the three major QTL we identify are still mapped to relatively broad genetic distances (6, 16, and 5 cM for Q1, Q2, and Q3, respectively, on the standard genetic map of *D. melanogaster*) that encompass hundreds of genes (Table 3). Q3 covers a particularly large physical distance as it resides over the centromere of the third chromosome where the rate of recombination is low. We anticipate being able to improve resolution and decrease the size of the genomic regions implicated by maintaining the AIL for many additional generations prior to QTL mapping and by adding markers to increase the number of informative recombination events across QTL intervals (see Macdonald and Long 2007).

### Comparing posterior lobe QTL mapped within and between *Drosophila* species

An important challenge in evolutionary genetics is to describe the relationship between intra- and interspecific genetic variation (see Nuzhdin and Reiwitch 2000). Using data from QTL experiments, we can ask whether the properties of loci contributing to trait variation within a species are similar to the properties of loci responsible for trait divergence between species. Such efforts have been used to suggest a shared genetic basis for floral trait variation within *Mimulus guttatus* and between *M. guttatus* and *M. nasutus*, as 11/16 intraspecific QTL map to the same locations in the interspecific cross (Fishman *et al.* 2002; Hall *et al.* 2006). Conversely, due to a lack of overlap between QTL mapped in intra- and interspecies crosses, current evidence suggests there is a qualitative difference in the genetic architecture of courtship song within *D. melanogaster* and between *D. simulans* and *D. sechellia* (Gleason *et al.* 2002; Gleason and Ritchie 2004).

The main result from our study is the identification of at least three moderate-effect QTL contributing to posterior lobe shape between a pair of inbred lines of *D. melanogaster*. The positions of these QTL map to approximately the same locations as QTL mapped in various interspecific crosses (Figure 7 in Liu *et al.* 1996; Figure 3 in Macdonald and Goldstein 1999; Figure 2 in Zeng *et al.* 2000; Figure 6 in Masly *et al.* 2011). Interspecific posterior lobe QTL have also been mapped to the tip of 2L and 3L in these studies, sites where we also find LOD scores just above the QTL significance threshold. This overlap in QTL positions suggests some of the same genes could be responsible for lobe shape variation both within and among species of *Drosophila*.

There is, of course, a clear caveat: Mapping resolution in all studies considered is relatively low and with >14,000 genes, only three major chromosomes, and the possibility that a large number of genes influence the trait, these QTL could overlap simply by chance. Short of positionally cloning the causative gene (see Wittkopp *et al.* 2009), progress toward a rigorous comparison of the pattern of genetic variation within and among species is likely to come only when the QTL are resolved to very short intervals and can be isolated from the effects of others in introgression lines. In general, large, highly recombinant mapping populations must be employed to achieve this, although in *D. melanogaster* investigators can make use of molecularly characterized deletions or loss-of-function mutations to implicate putative causative genes via quantitative complementation tests (Long *et al.* 1996; Pasyukova *et al.* 2000).

### Candidate gene analysis

In common with the rest of the male and female adult genitalia, the posterior lobe develops from the larval genital imaginal disc. Chatterjee *et al.* (2011) used microarrays to identify 22 euchromatic genes that consistently differ in expression between male and female *D. melanogaster* genital discs across three developmental time points (L3 larvae, 6 hr and 20 hr after puparium formation). Seven of these genes were also found by Masly *et al.* (2011), comparing male and female discs in L3 larvae only. In addition, at least 2 of the genes found in both studies (*Pox neuro* and *Drop*) can be mutated to alter adult posterior lobe morphology (Boll and Noll 2002; Chatterjee *et al.* 2011). We highlight the positions of these 22 loci in Figure 4 (red points above each plot) and note a visually striking overlap between the mPC1 QTL peaks and candidate gene positions, particularly on the autosomes. Interestingly, the classic sex-determining gene *doublesex* (Hildreth 1965) is within the 2-LOD drop for the pericentromeric QTL Q3. This gene was identified as male-biased in the developing *D. melanogaster* genital disc by both Chatterjee *et al.* (2011) and Masly *et al.* (2011), and it was also one of the genes the latter identified as differentially expressed between *D. mauritiana* and *D. sechellia* in male genital discs.

To test for a statistical association between our QTL results and these candidate loci, we used a resampling procedure (Keightley *et al.* 1998). One million sets of 18 autosomal loci were randomly sampled, and the mPC1 LOD scores at the 18 positions summed (the X chromosome was ignored because the low *Sam* allele frequency on this chromosome in the F_17_ likely compromised mapping power). This procedure gives a distribution of the expected LOD scores assuming no relationship between our phenotype and the Chatterjee *et al.* (2011) candidate genes. The sum of the PC1 LOD scores at the actual locations of the 18 autosomal candidate genes is 68.7, which is in the top 1% of the null distribution (mean = 39.1, standard deviation = 9.33), indicating a significant association between the two datasets. Thus, those genes that show sex-biased gene expression in genital discs and are present within QTL intervals (see Figure 4 legend) are plausible candidates to harbor natural genetic variants contributing to posterior lobe shape.

## Supplementary Material

Supporting Information
