# Supplementary material for: Multiple Quantitative Trait Loci Influence the Shape of a Male-Specific Genital Structure in Drosophila melanogaster
Source: G3 (Bethesda). 2011 Oct 1;1(5):343–51. doi: 10.1534/g3.111.000661 (PMC3276151; doi:10.1534/g3.111.000661)
Supplement: Supporting Information [file supp_1_5_343__index.html]

Supporting Information 

# Multiple Quantitative Trait Loci Influence the Shape of a Male-Specific Genital Structure in *Drosophila melanogaster*

## Supporting Information for McNeil, Bain, and Macdonald, 2011

**Files in this Data Supplement:**

- Supporting Information - Figures S1-S4 and Files S1-S3 (PDF, 1 MB)
- Figure S1 - Posterior lobes from 15 *D. melanogaster* inbred lines (PDF, 216 KB)
- Figure S2 - Outlines of lobes from different genotypes showing the change in shape associated with the mPC1 measure (PDF, 360 KB)
- Figure S3 - Coarse- and fine-mapping likelihood profiles for all traits (PDF, 236 KB)
- Figure S4 - Frequency of the *Sam* allele at markers in the mapping panels (PDF, 52 KB)
- File S1 - Development of SNP markers (.xls, 48 KB)
- File S2 - Raw phenotypes and genotypes for all F2 individuals (.csv, 140 KB)
- File S3 - Raw phenotypes and genotypes for all F17 individuals (.csv, 100 KB)
