# Supplementary material for: Multiple Quantitative Trait Loci Influence the Shape of a Male-Specific Genital Structure in Drosophila melanogaster
Source: G3 (Bethesda). 2011 Oct 1;1(5):343–51. doi: 10.1534/g3.111.000661 (PMC3276151; doi:10.1534/g3.111.000661)
Supplement: Supporting Information [file supp_1.5.343_000661.pdf]

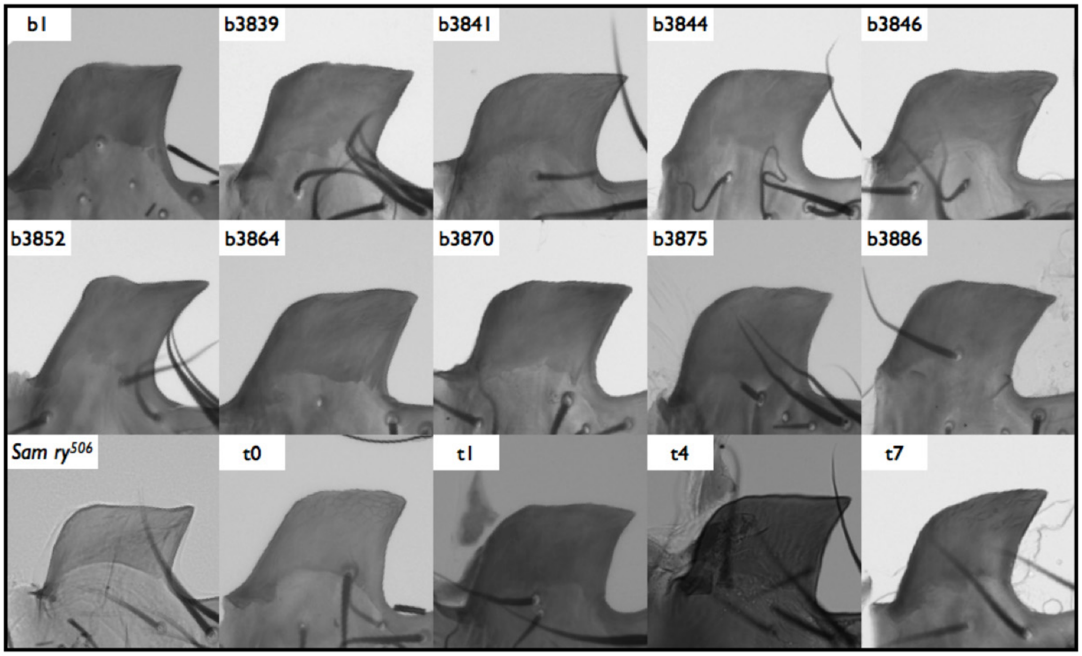

Figure S1 Posterior lobes from 15 *D. melanogaster* inbred lines. A single, representative lobe is shown for each of the strains used in the study: b1, b3839, b3841, b3844, b3846, b3852 (mapping strain), b3864, b3870, b3875, b3886, *Sam ry*^{506} (mapping strain), t0, t1, t4, and t7. All images were taken at the same magnification.

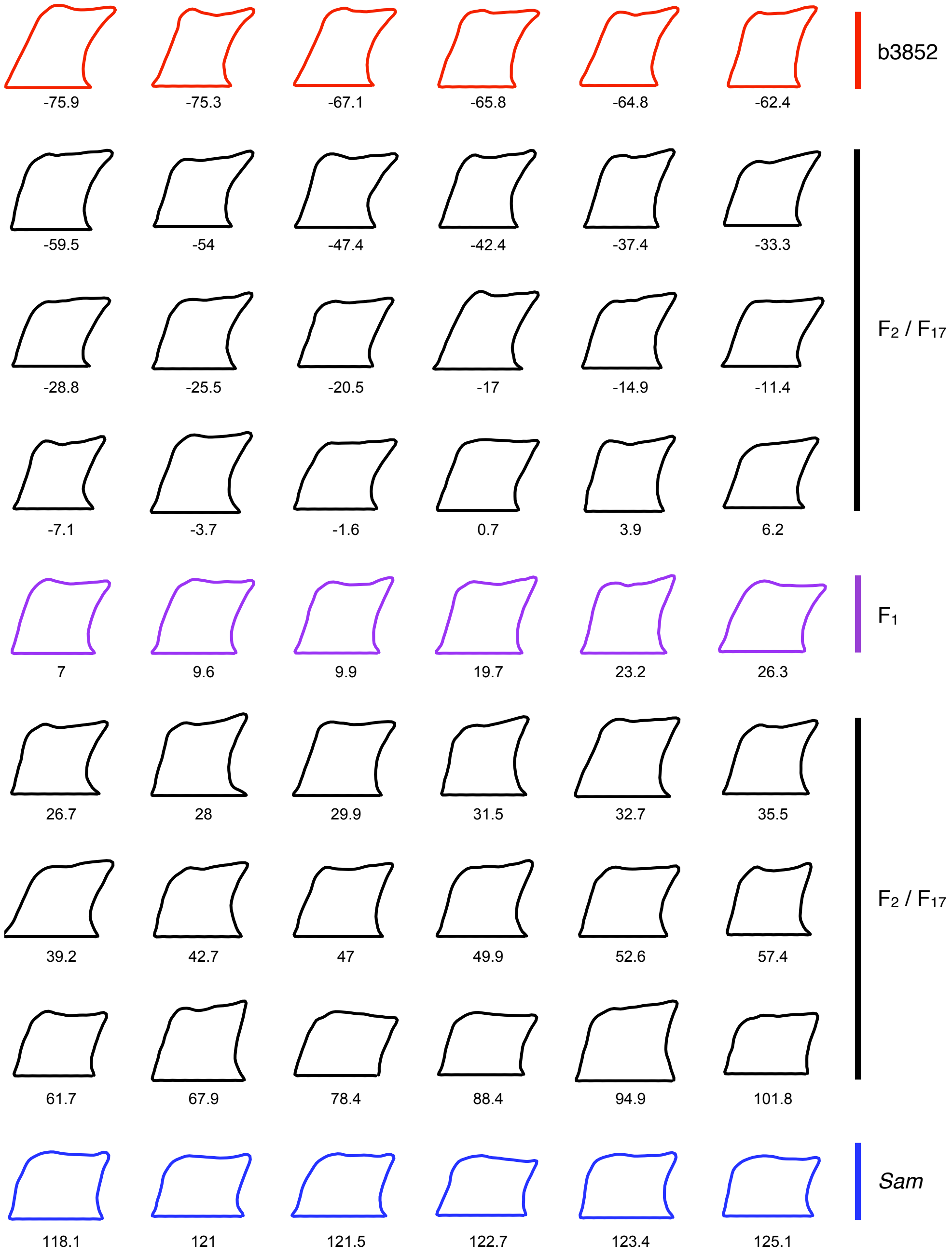

Figure S2 Outlines of lobes from different genotypes showing the change in shape associated with the mPC1 measure. Lobes from a subset of b3852 (red), *Sam* (blue), F_1 (purple), and recombinant F_2 or F_{17} individuals (black) are shown, sorted by their mPC1 score (x 10^{-4}).

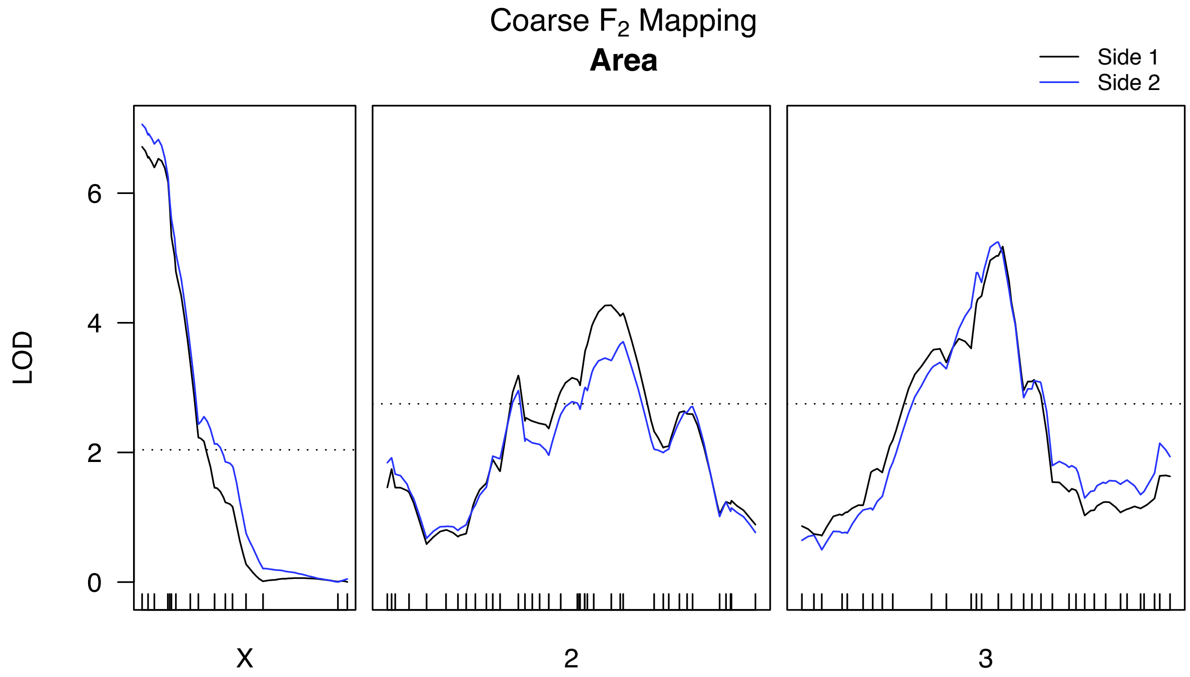

Coarse F_2 Mapping
Area
Side 1
Side 2
LOD
0
2
4
6
X
2
3

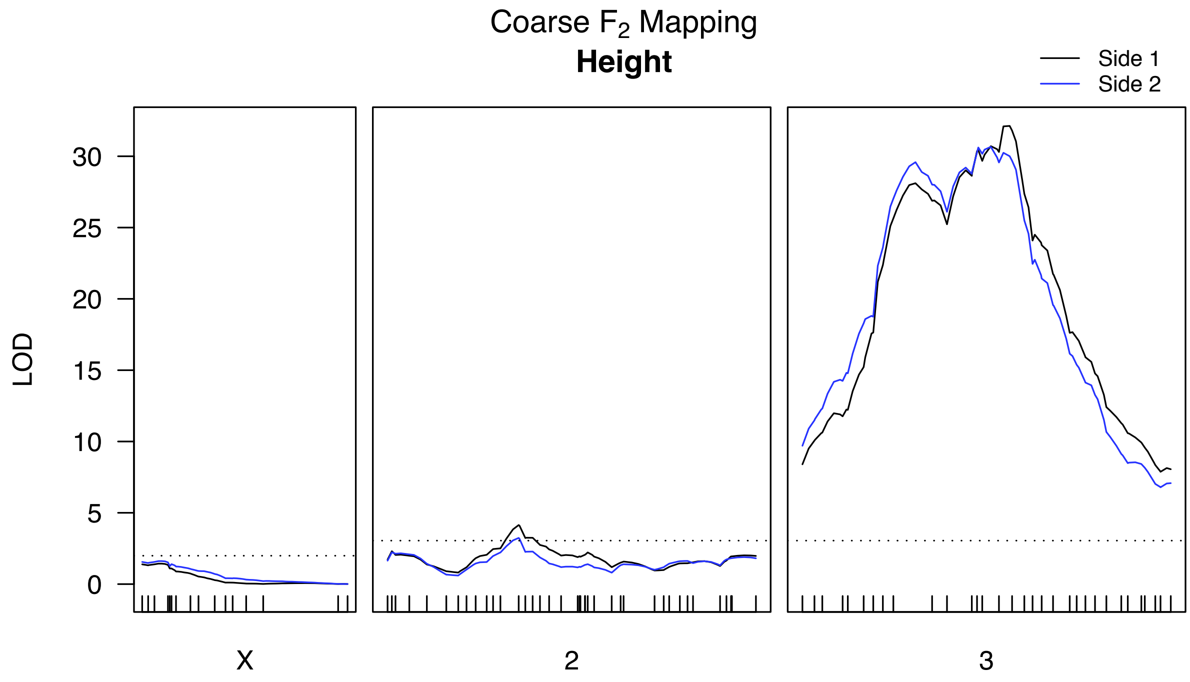

Coarse F_2 Mapping
Height
Side 1
Side 2
LOD
0
5
10
15
20
25
30
X
2
3

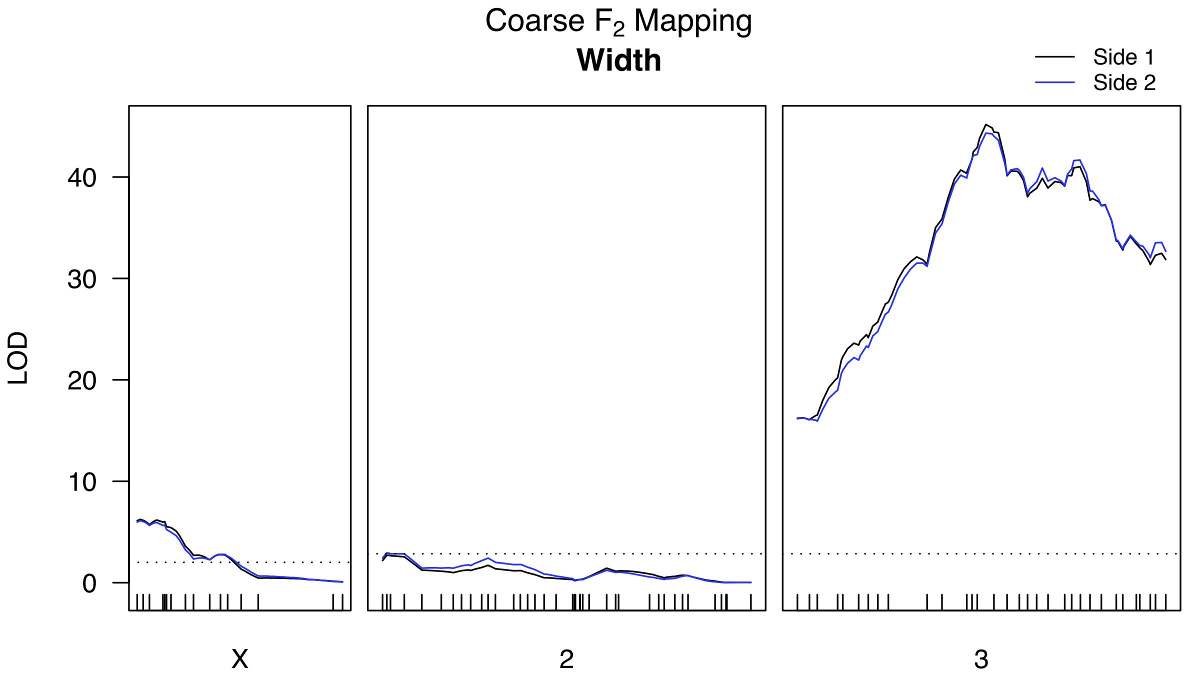
Coarse F_2 Mapping
Width
Side 1
Side 2
LOD
0
10
20
30
40
X
2
3

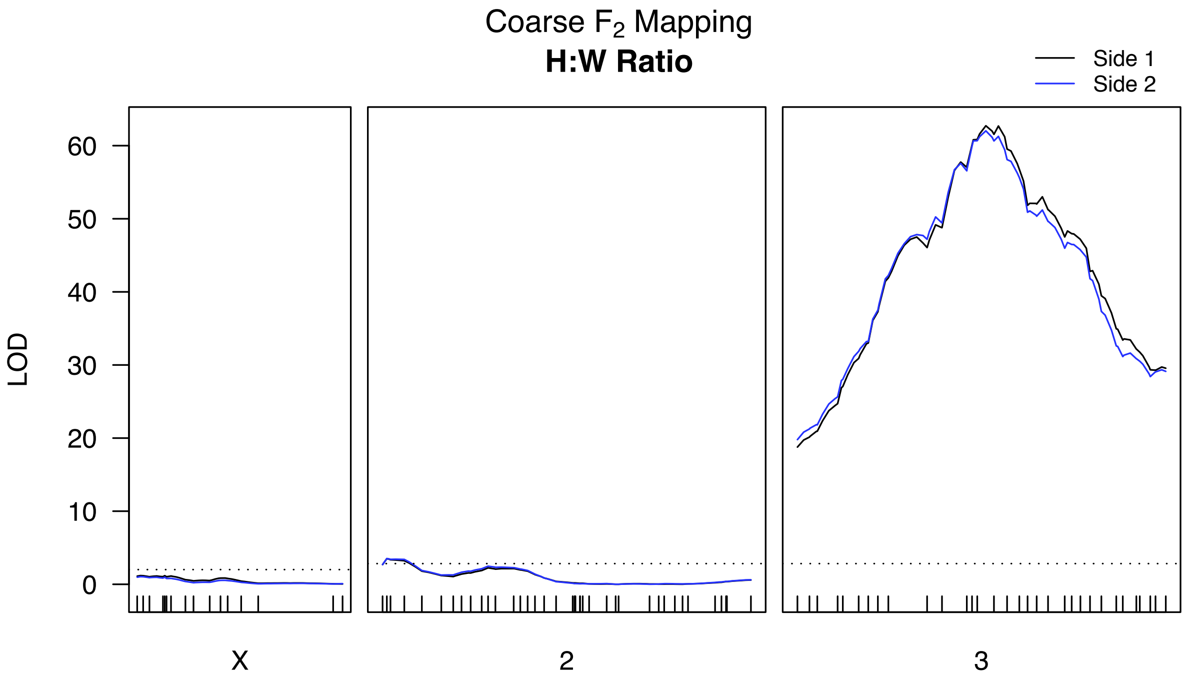
Coarse F_2 Mapping
H:W Ratio
Side 1
Side 2
LOD
0
10
20
30
40
50
60
X
2
3

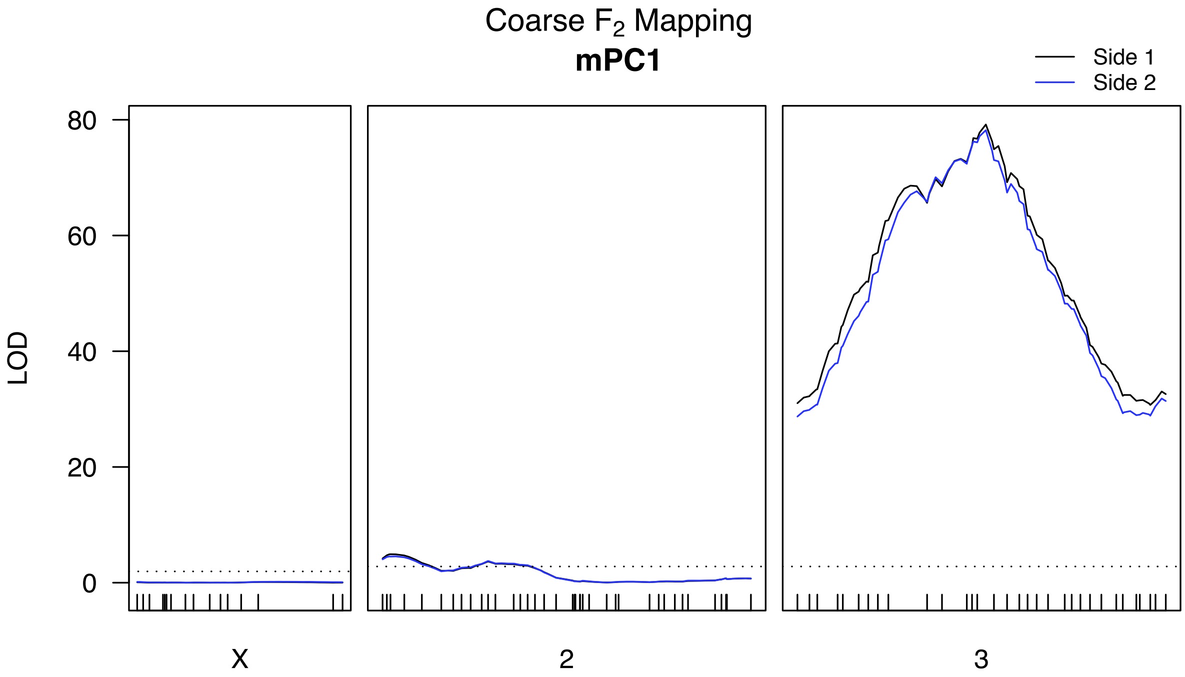
Coarse F_2 Mapping
mPC1
Side 1
Side 2
LOD
80
60
40
20
0
X
2
3

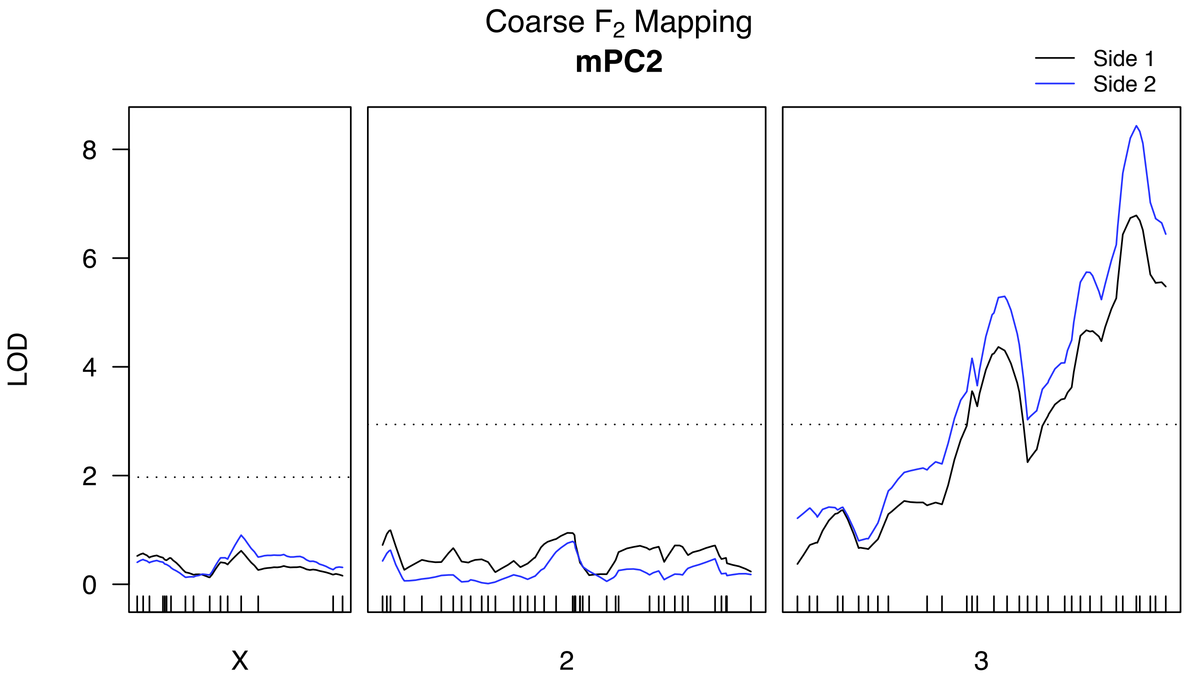
Coarse F_2 Mapping
mPC2
Side 1
Side 2
LOD
8
6
4
2
0
X
2
3

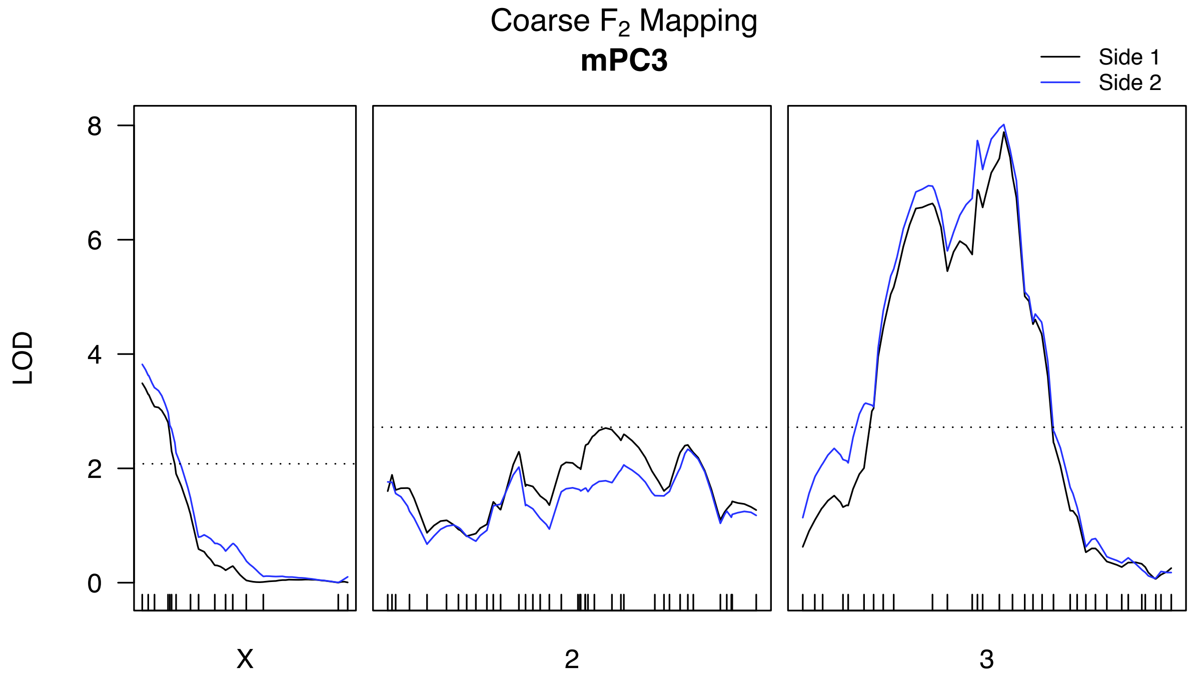
Coarse F_2 Mapping
mPC3
Side 1
Side 2
LOD
8
6
4
2
0
X
2
3

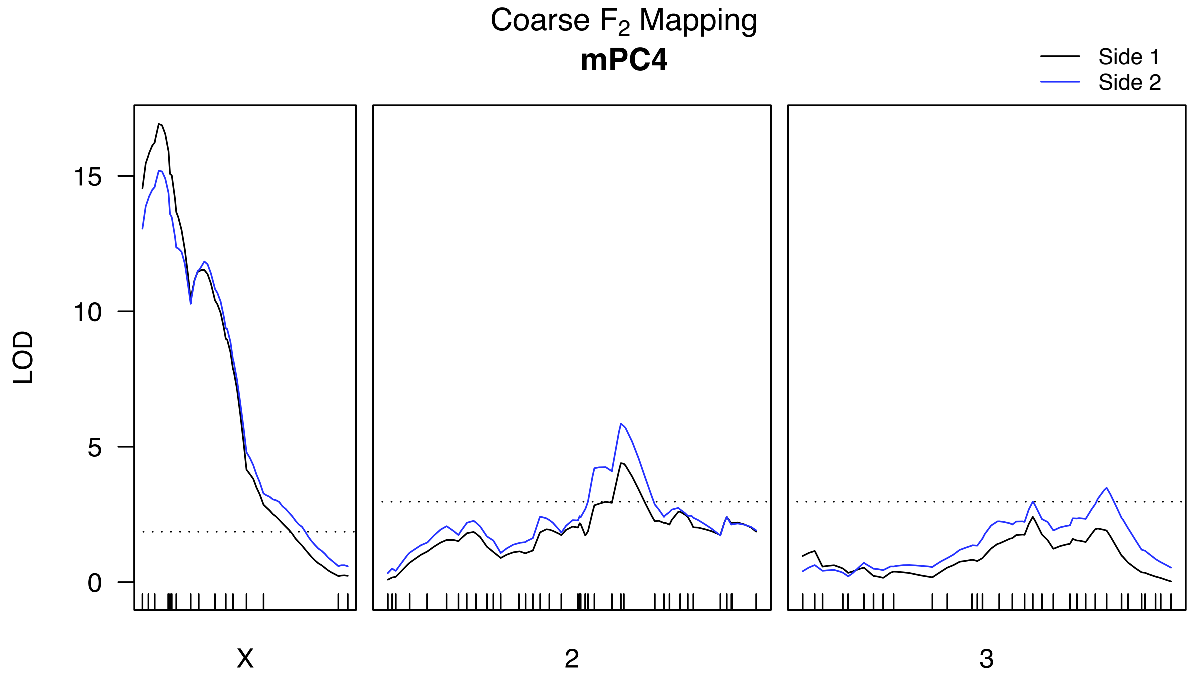
Coarse F_2 Mapping
mPC4
Side 1
Side 2
LOD
15
10
5
0
X
2
3

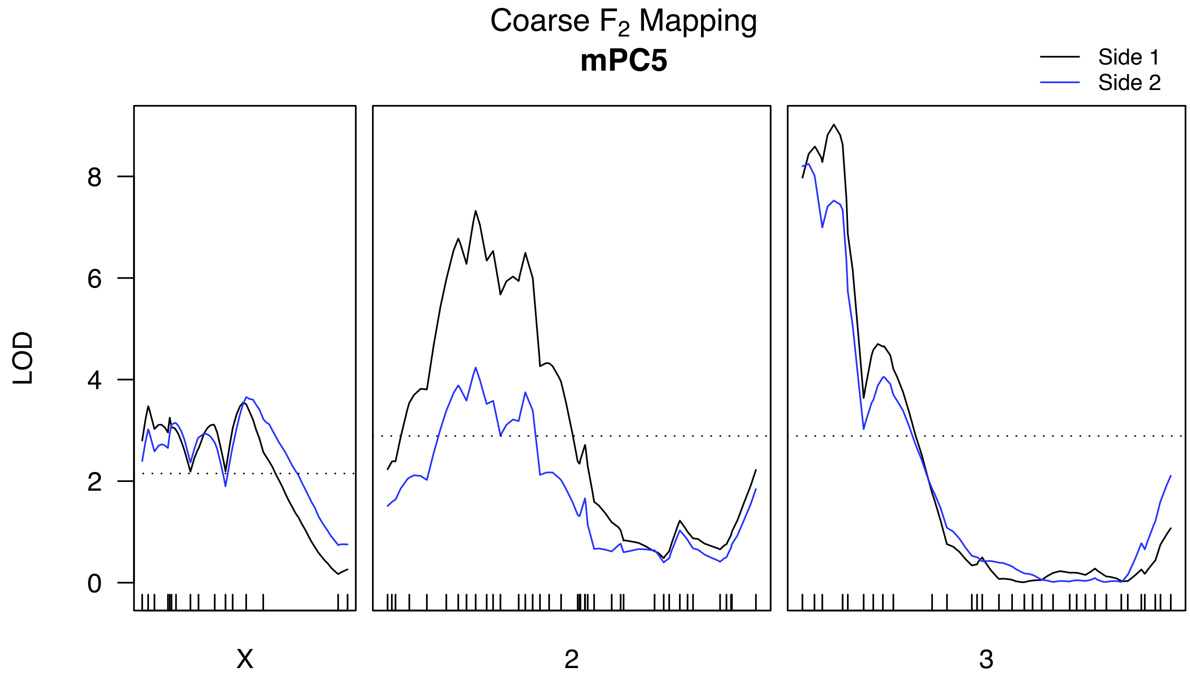
Coarse F_2 Mapping
mPC5
Side 1
Side 2
LOD
0
2
4
6
8
X
2
3

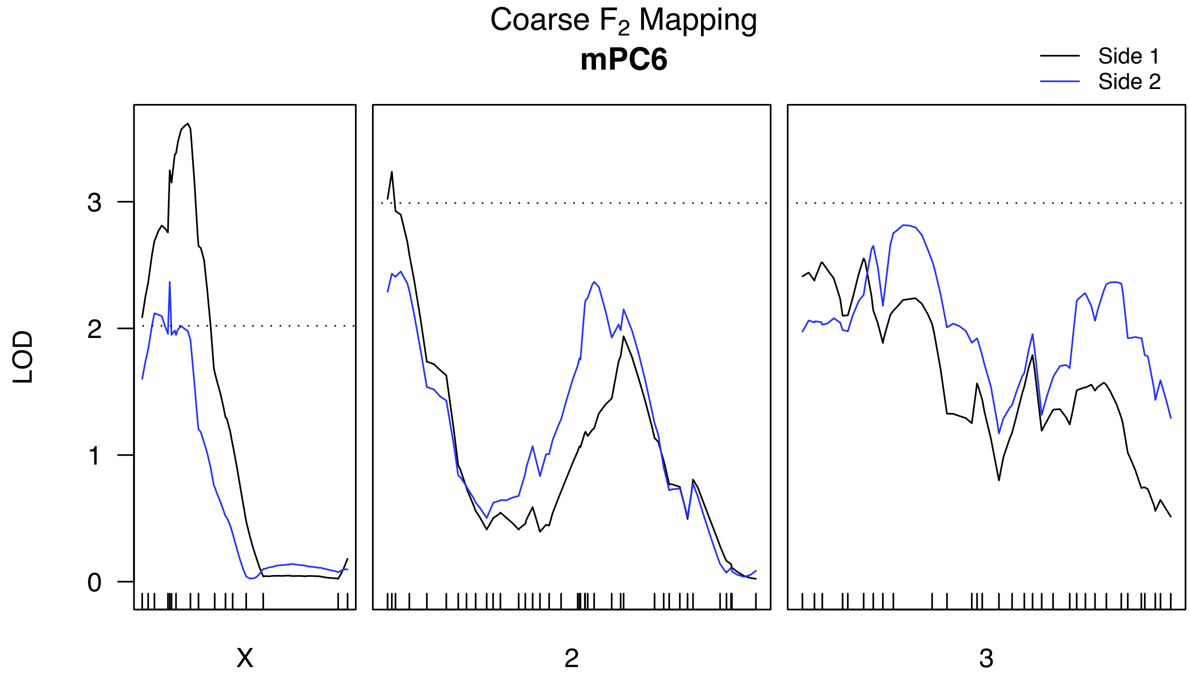
Coarse F_2 Mapping
mPC6
Side 1
Side 2
LOD
0
1
2
3
X
2
3

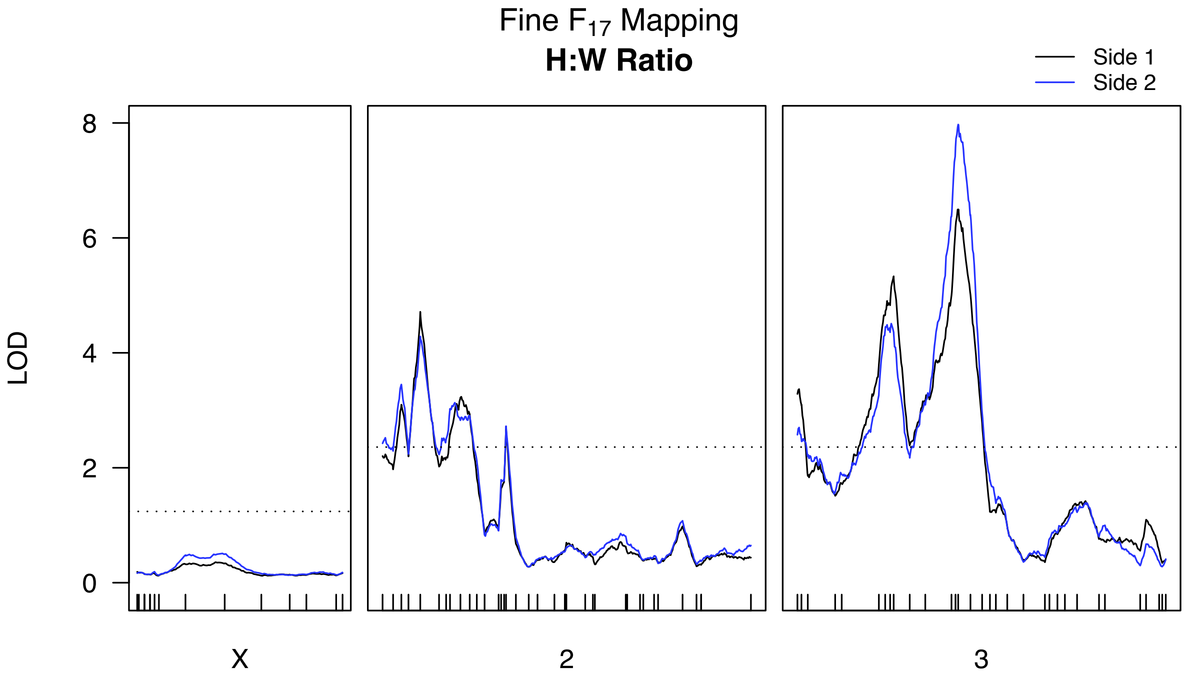

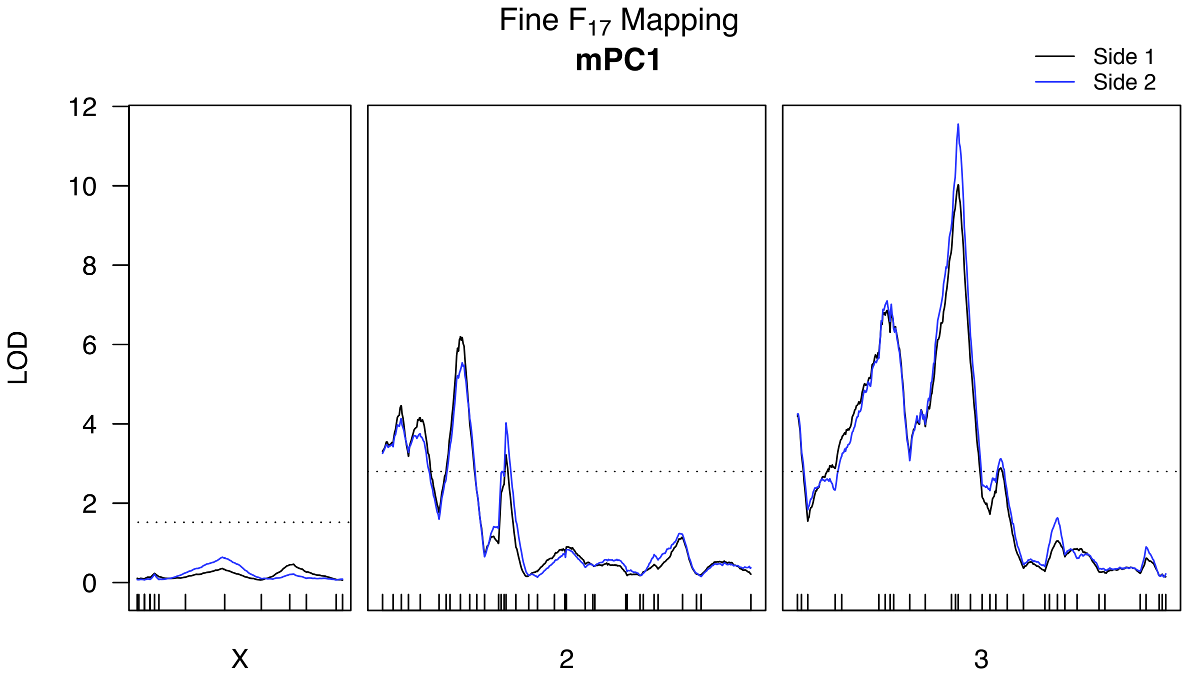

Figure S3 Coarse- and fine-mapping likelihood profiles for all traits. Each panel shows the results of interval mapping (IM) for a given phenotype, and the form of the plots is similar to those in Figure 4. Two curves are provided to demonstrate that similar results are generated no matter which of the two lobes is measured for a given fly. Note that the scale of the *y*-axis differs across plots.

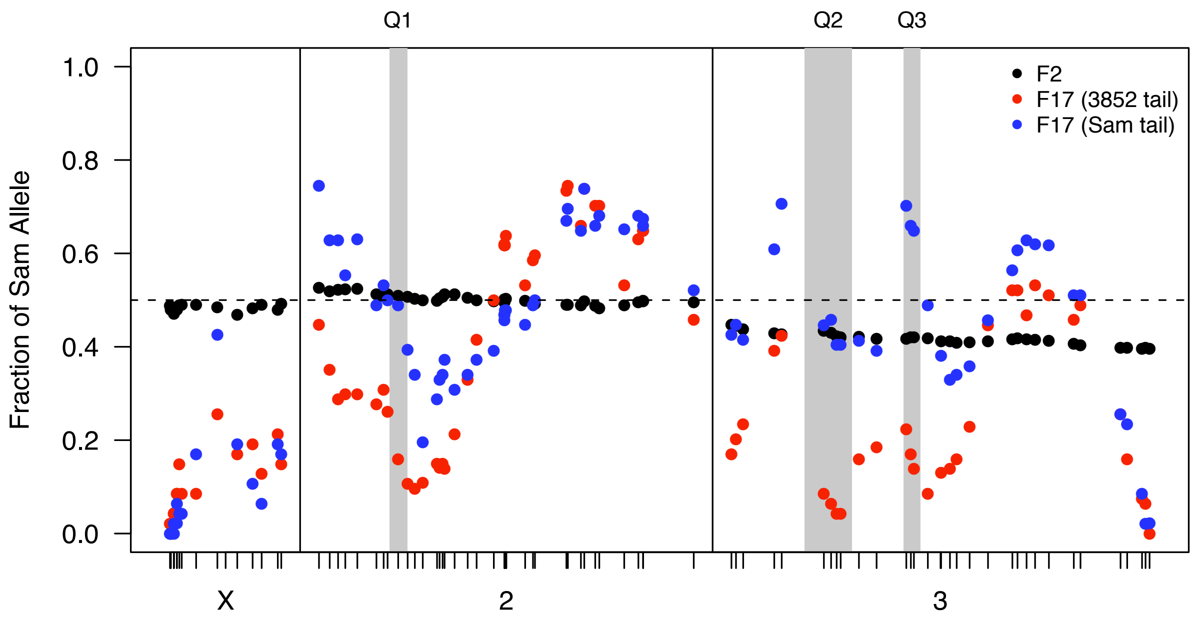

Figure S4 Frequency of the *Sam* allele at markers in the mapping panels. For every marker we directly count the fraction of alleles coming from the *Sam* parental line in each set of genotyped individuals - F_2 (black), b3852-like F_{17} tail (red), and *Sam*-like F_{17} tail (blue). The frequencies are plotted against the marker positions on the expanded F_{17} genetic map, and the three major QTL intervals are marked with gray boxes. In the F_2, *Sam* alleles are typically close to the expected frequency of 0.5, although frequencies are slightly lower for chromosome 3, potentially due to negative fitness consequences associated with the mutant *ry*^{506} allele. In the F_{17} both the X chromosome and the telomeric end of chromosome 3R show a dearth of *Sam* alleles in both tail samples, indicating mapping power in these regions is likely to be poor.

Files S1 – S3

Supporting Data

Files S1-S3 are available for download at http://www.g3journal.org/lookup/suppl/doi:10.1534/g3.111.000661/-/DC1.

File S1 Development of SNP markers. The nucleotide sequence surrounding each SNP is provided, along with the position of the SNP in the *D. melanogaster* reference genome (release 5.2), and the GoldenGate assay genotyping score provided by Illumina.

File S2 Raw phenotypes and genotypes for all F2 individuals. All phenotypes discussed in the text are provided for each individual, along with their genotypes (A = homozygous b3852 genotype, H = heterozygote, B = homozygous *Sam* genotype). Genetic positions estimated via *R/qtl* are also provided for all markers. This file is suitable for direct input into *R/qtl*.

File S3 Raw phenotypes and genotypes for all F17 individuals. See legend for File S2.
