# Supplementary material for: Multiple Quantitative Trait Loci Influence the Shape of a Male-Specific Genital Structure in Drosophila melanogaster
Source: G3 (Bethesda). 2011 Oct 1;1(5):343–51. doi: 10.1534/g3.111.000661 (PMC3276151; doi:10.1534/g3.111.000661)
Supplement: Supporting Information [file supp_1.5.343_FigureS1.pdf]

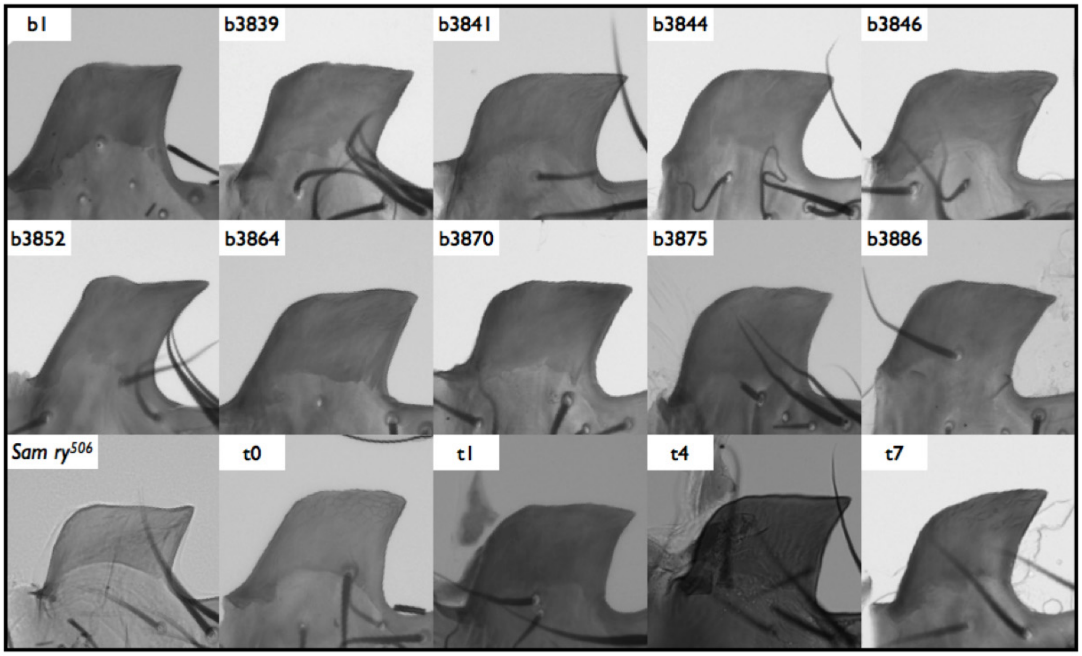

Figure S1 Posterior lobes from 15 *D. melanogaster* inbred lines. A single, representative lobe is shown for each of the strains used in the study: b1, b3839, b3841, b3844, b3846, b3852 (mapping strain), b3864, b3870, b3875, b3886, *Sam ry*^{506} (mapping strain), t0, t1, t4, and t7. All images were taken at the same magnification.
