# Supplementary material for: Multiple Quantitative Trait Loci Influence the Shape of a Male-Specific Genital Structure in Drosophila melanogaster
Source: G3 (Bethesda). 2011 Oct 1;1(5):343–51. doi: 10.1534/g3.111.000661 (PMC3276151; doi:10.1534/g3.111.000661)
Supplement: Supporting Information [file supp_1.5.343_FigureS2.pdf]

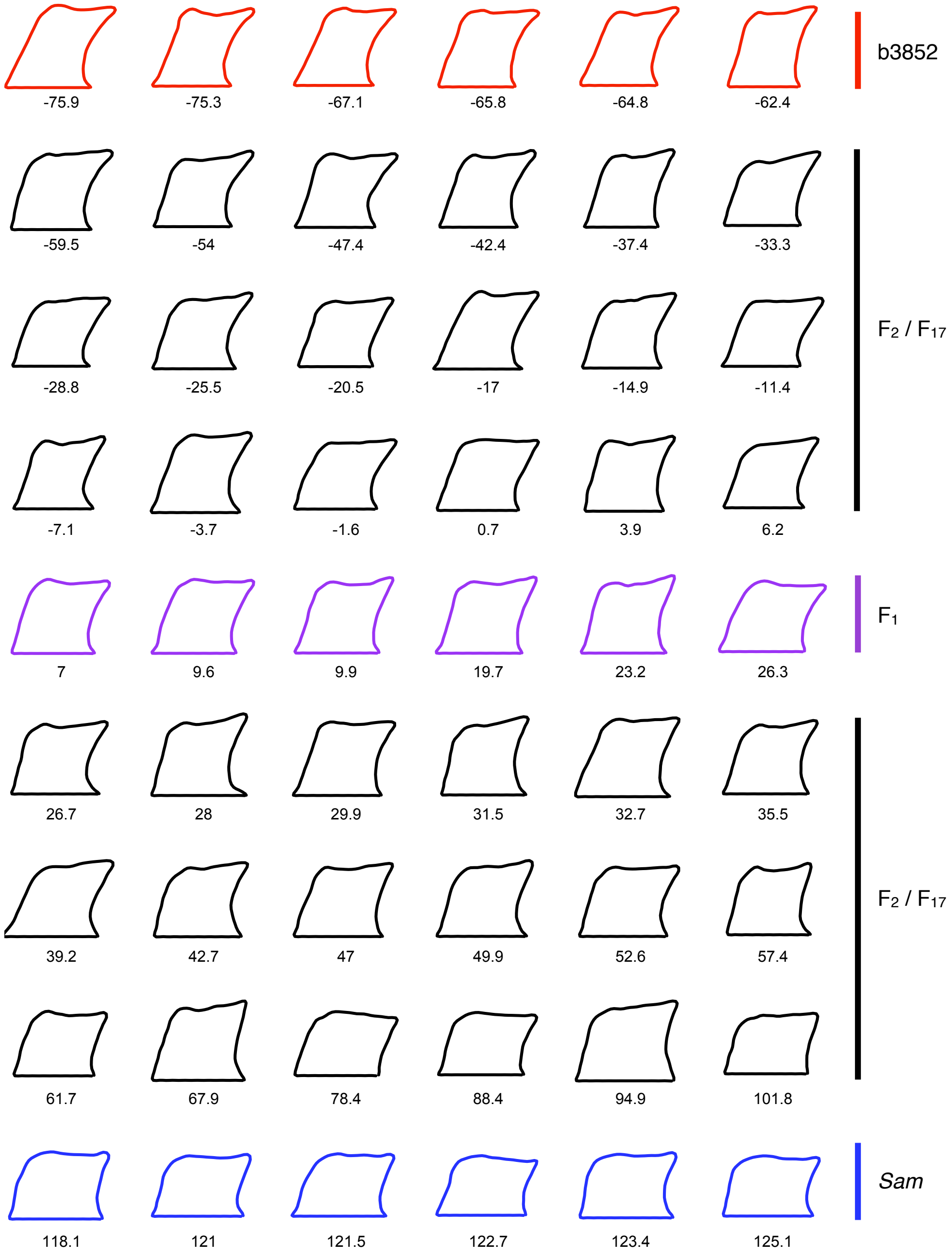

Figure S2 Outlines of lobes from different genotypes showing the change in shape associated with the mPC1 measure. Lobes from a subset of b3852 (red), *Sam* (blue), F_1 (purple), and recombinant F_2 or F_{17} individuals (black) are shown, sorted by their mPC1 score (x 10^{-4}).
