# Supplementary material for: Multiple Quantitative Trait Loci Influence the Shape of a Male-Specific Genital Structure in Drosophila melanogaster
Source: G3 (Bethesda). 2011 Oct 1;1(5):343–51. doi: 10.1534/g3.111.000661 (PMC3276151; doi:10.1534/g3.111.000661)
Supplement: Supporting Information [file supp_1.5.343_FigureS3.pdf]

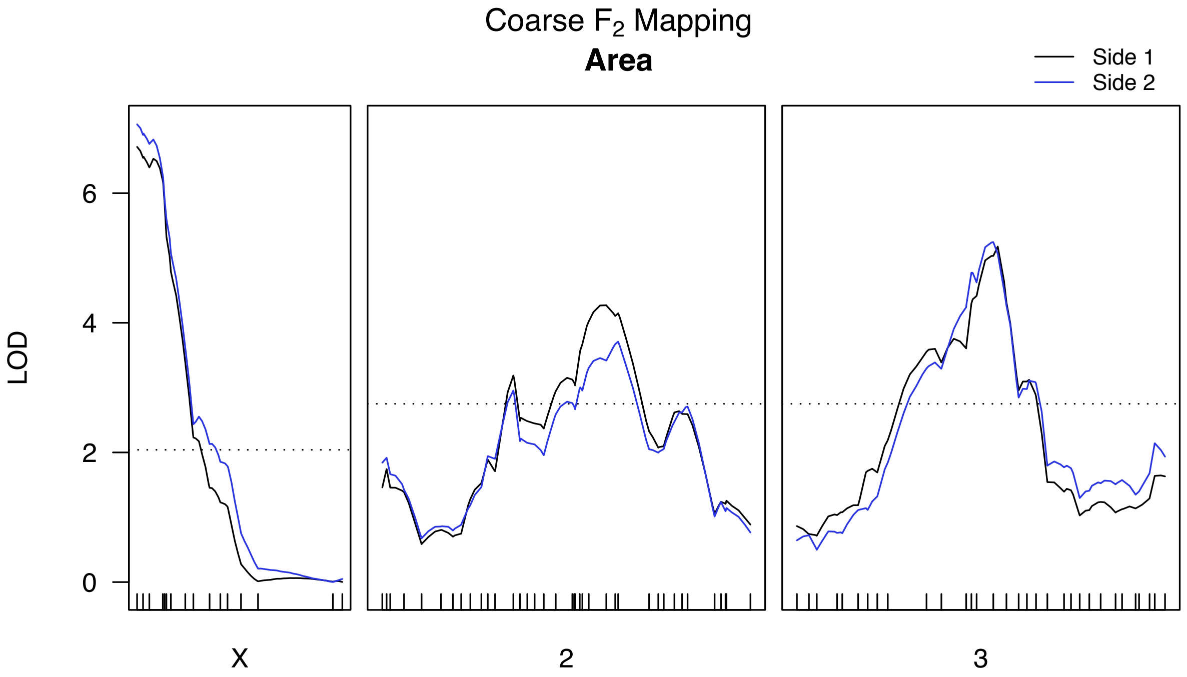
Coarse F2 Mapping
Area
Side 1
Side 2
LOD
0
2
4
6
X
2
3

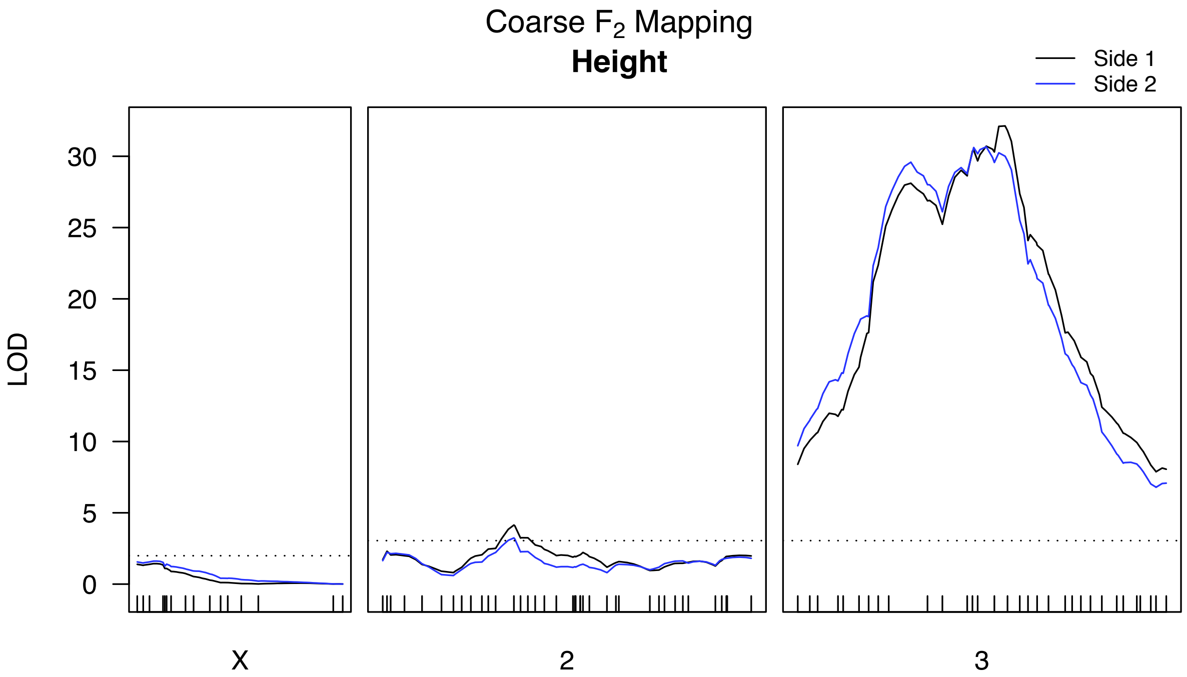
Coarse F2 Mapping
Height
Side 1
Side 2
LOD
0
5
10
15
20
25
30
X
2
3

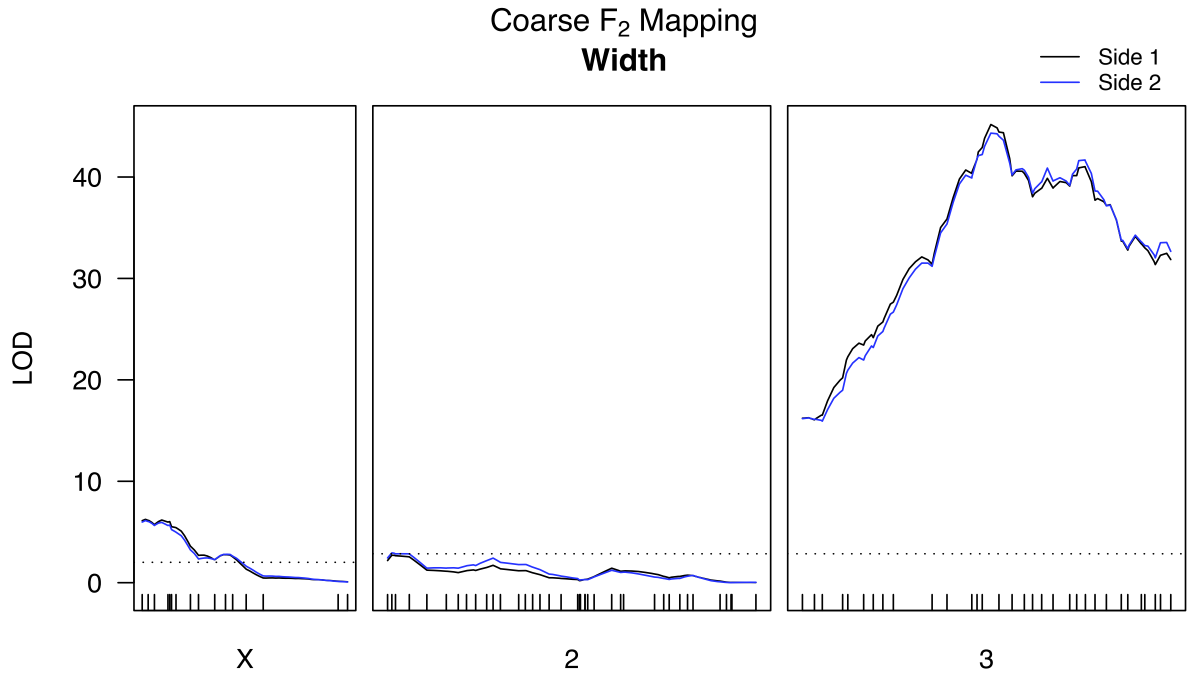
Coarse F_2 Mapping
Width
Side 1
Side 2
LOD
0
10
20
30
40
X
2
3

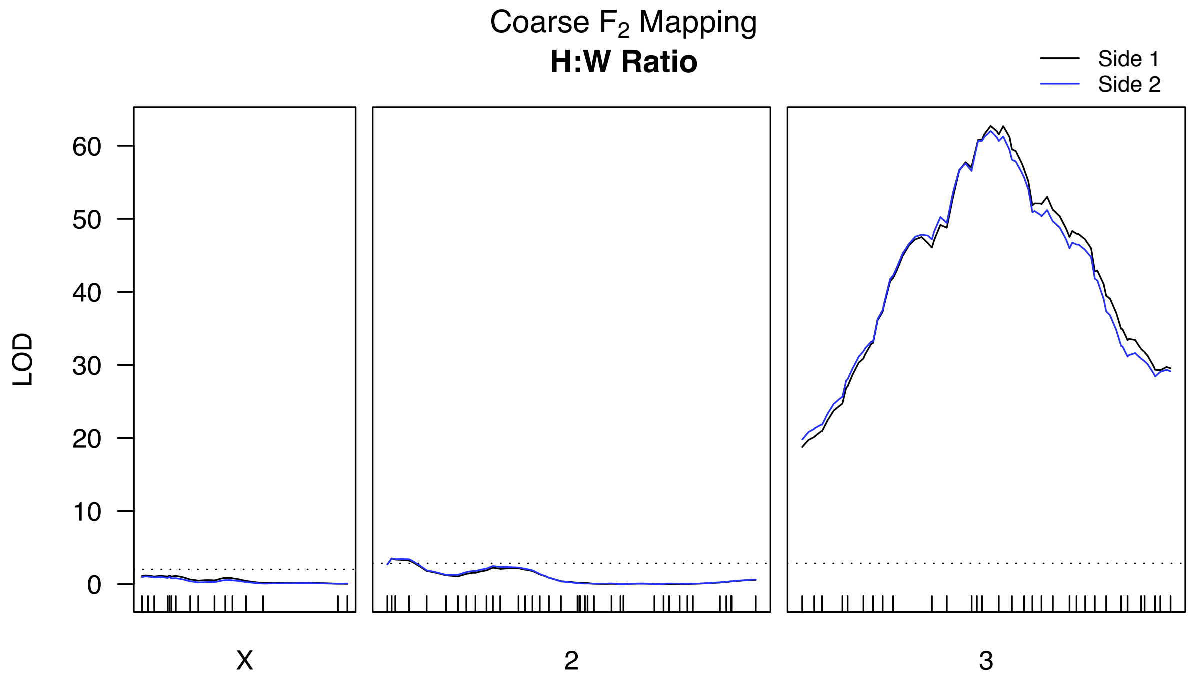
Coarse F_2 Mapping
H:W Ratio
Side 1
Side 2
LOD
0
10
20
30
40
50
60
X
2
3

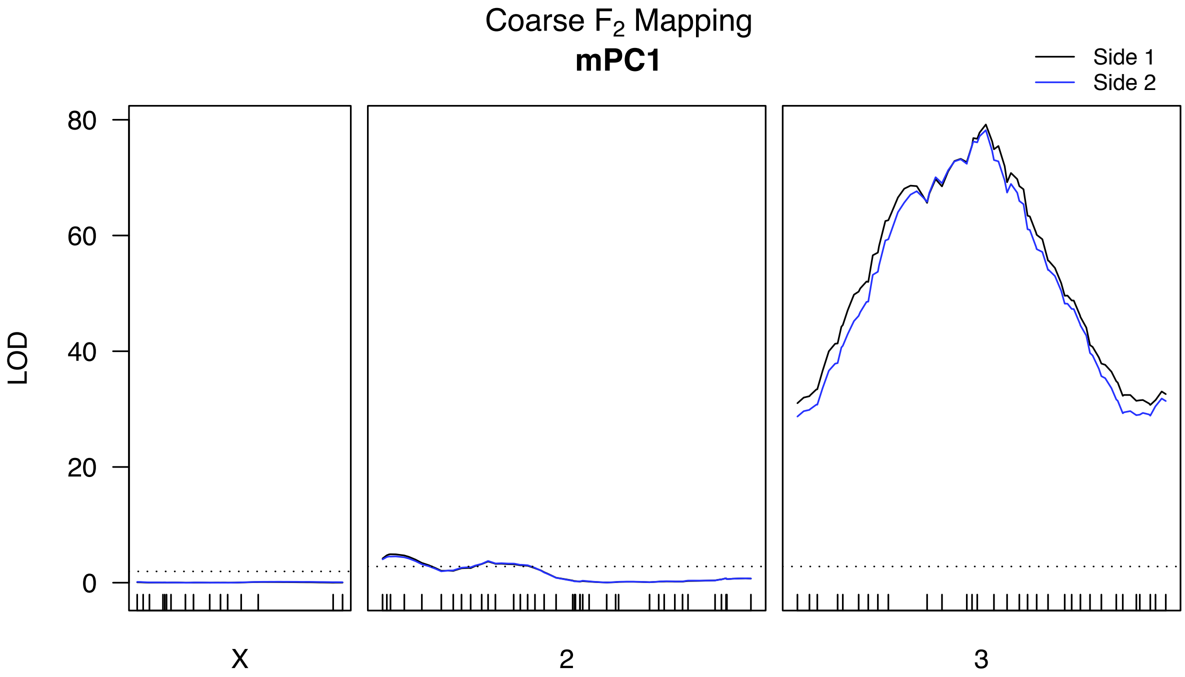

Coarse F_2 Mapping
mPC1
Side 1
Side 2
LOD
80
60
40
20
0
X
2
3

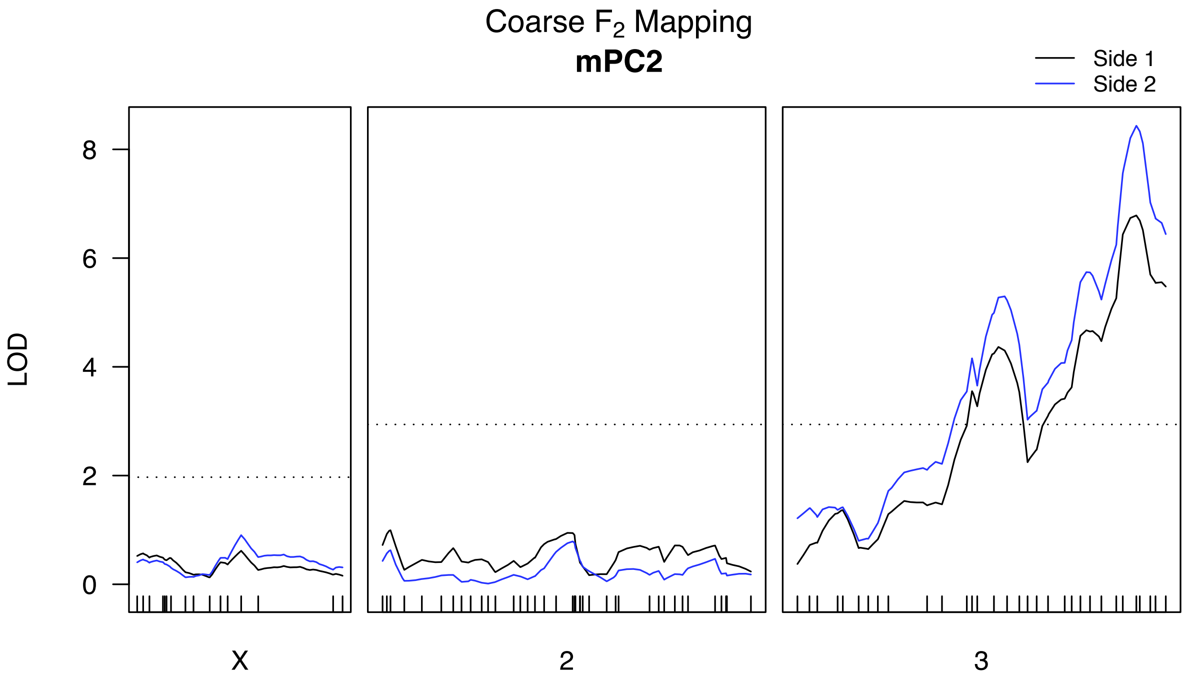

Coarse F_2 Mapping
mPC2
Side 1
Side 2
LOD
8
6
4
2
0
X
2
3

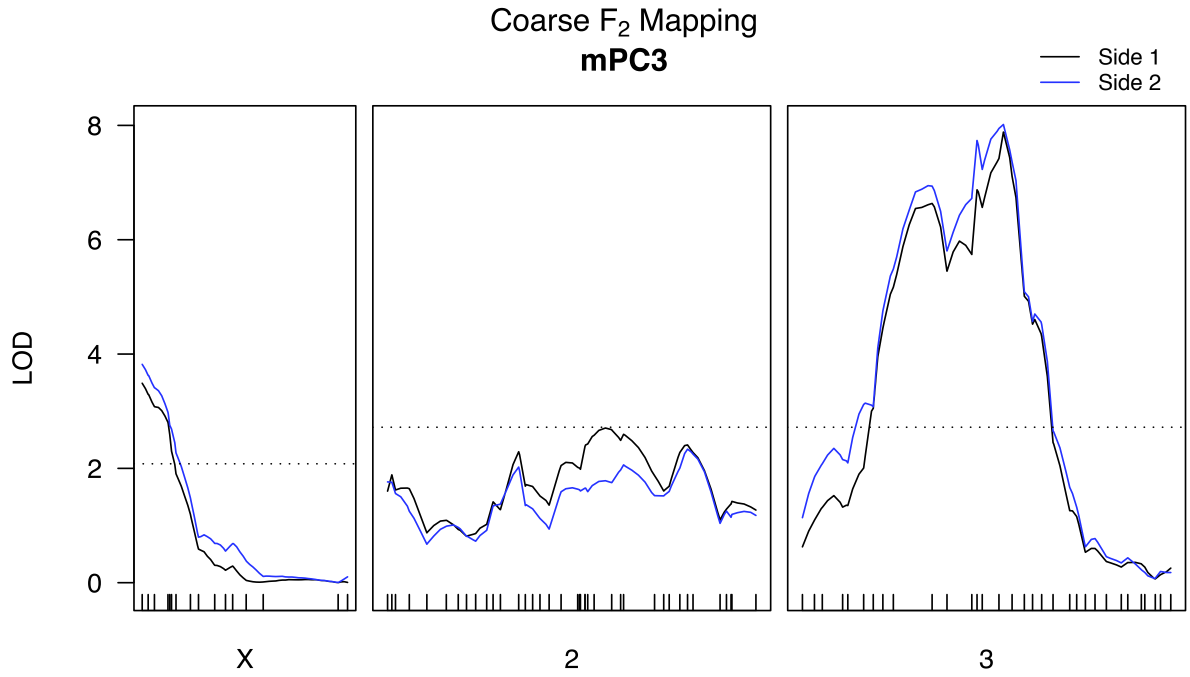
Coarse F_2 Mapping
mPC3
Side 1
Side 2
LOD
8
6
4
2
0
X
2
3

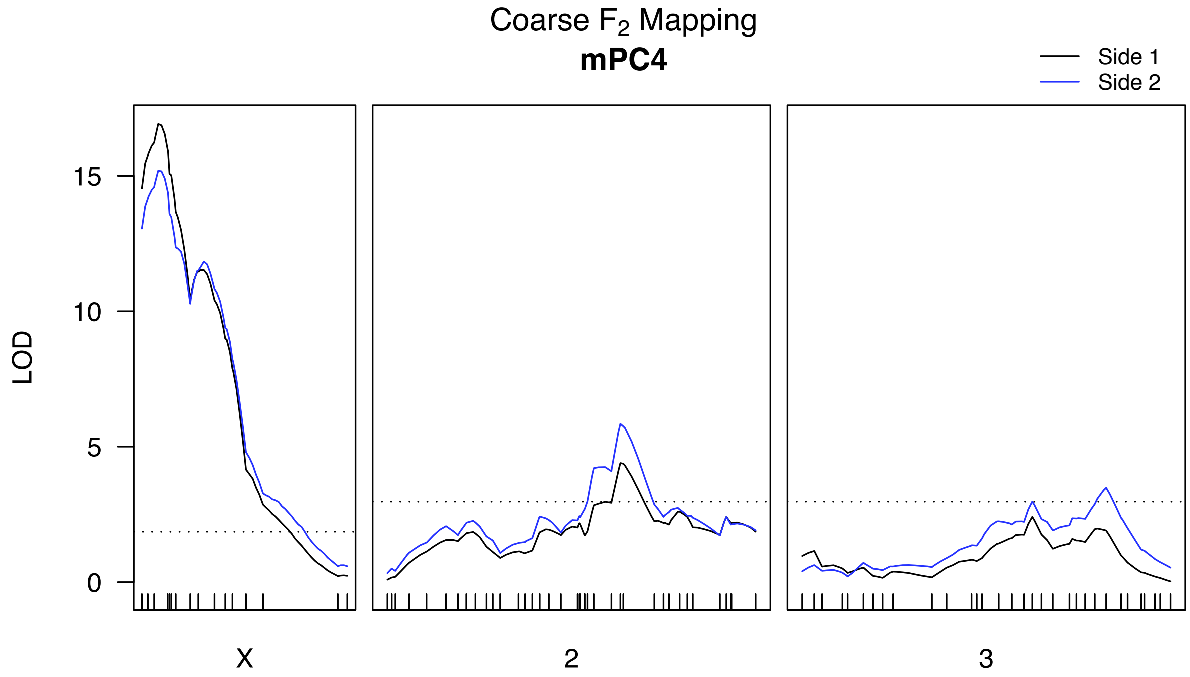
Coarse F_2 Mapping
mPC4
Side 1
Side 2
LOD
15
10
5
0
X
2
3

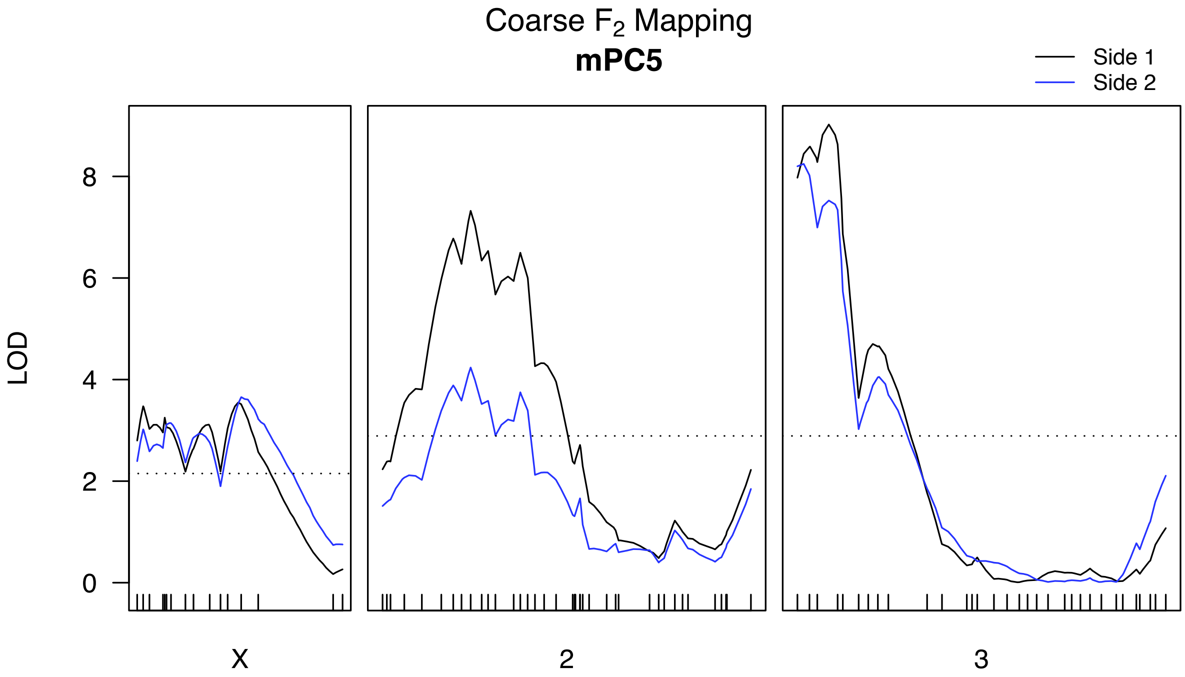
Coarse F_2 Mapping
mPC5
Side 1
Side 2
LOD
0
2
4
6
8
X
2
3

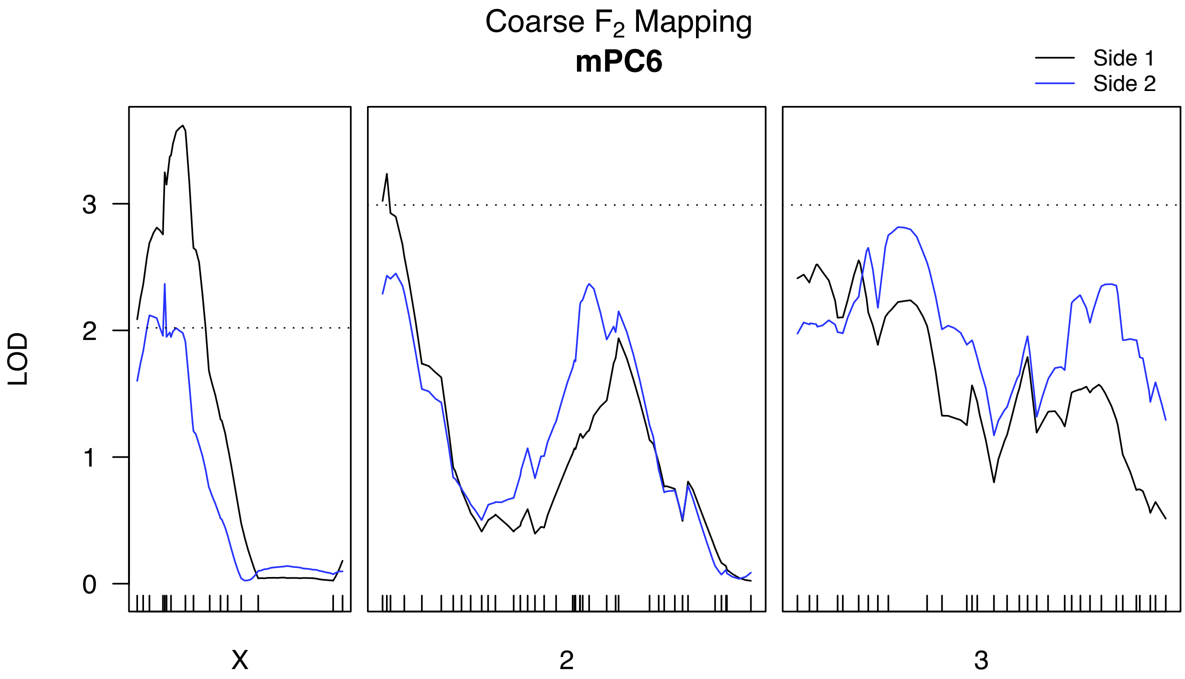
Coarse F_2 Mapping
mPC6
Side 1
Side 2
LOD
0
1
2
3
X
2
3

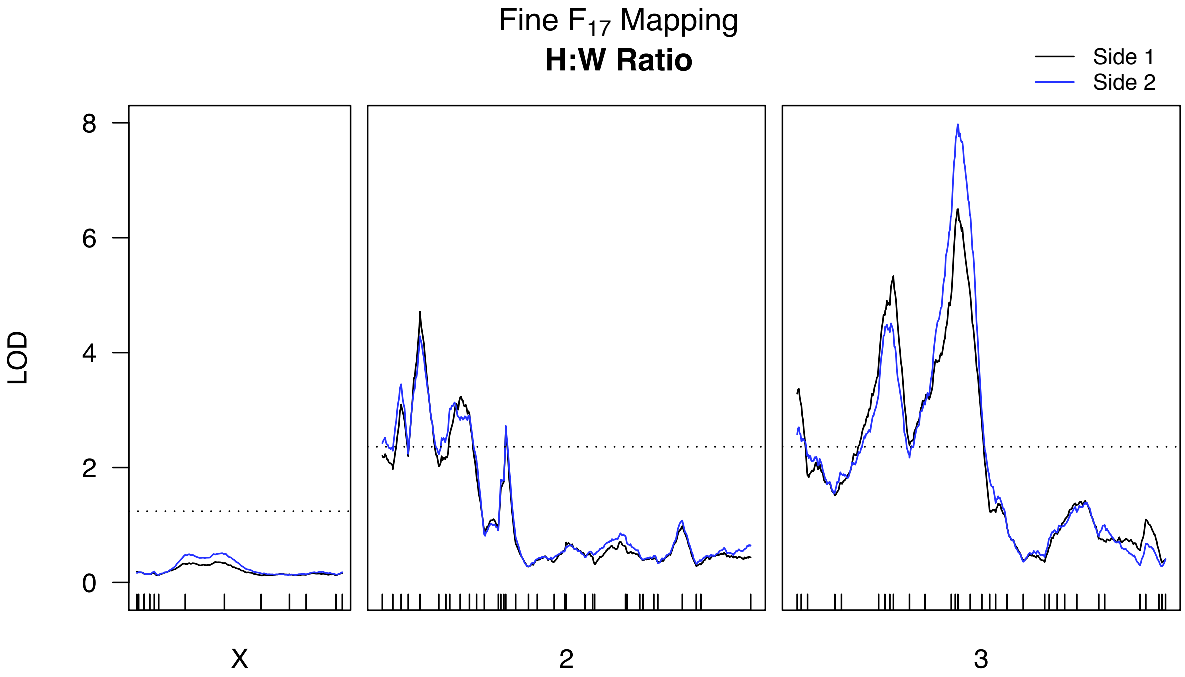

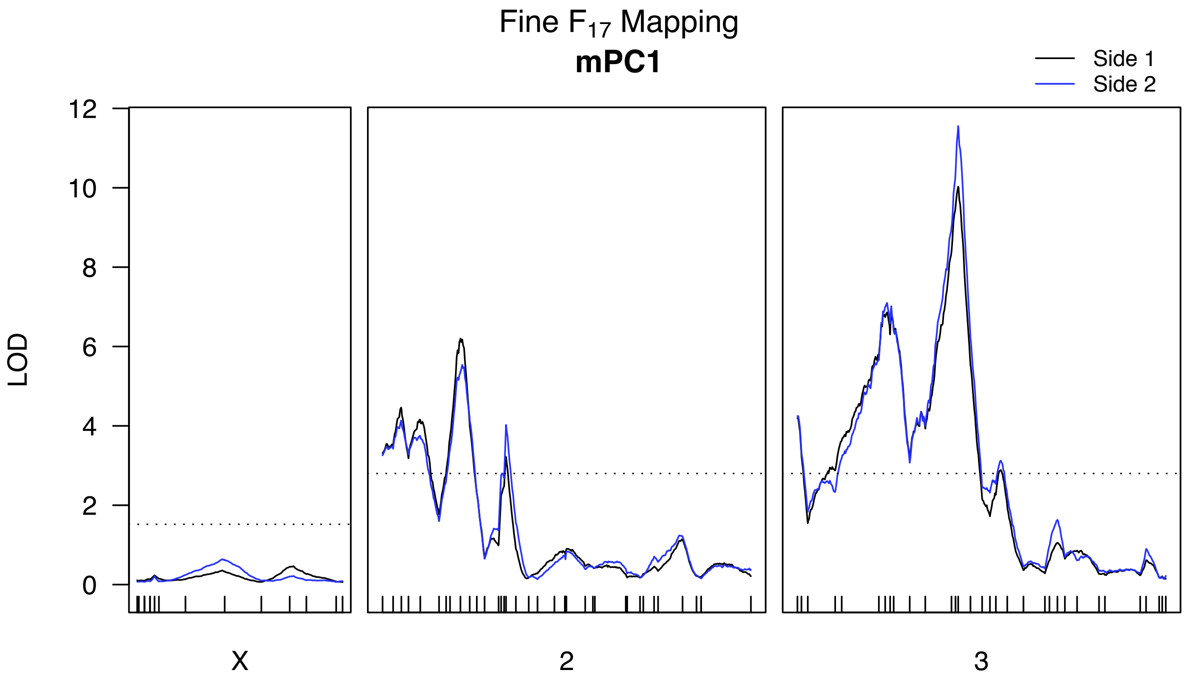

Figure S3 Coarse- and fine-mapping likelihood profiles for all traits. Each panel shows the results of interval mapping (IM) for a given phenotype, and the form of the plots is similar to those in Figure 4. Two curves are provided to demonstrate that similar results are generated no matter which of the two lobes is measured for a given fly. Note that the scale of the *y*-axis differs across plots.
