# Supplementary material for: Multiple Quantitative Trait Loci Influence the Shape of a Male-Specific Genital Structure in Drosophila melanogaster
Source: G3 (Bethesda). 2011 Oct 1;1(5):343–51. doi: 10.1534/g3.111.000661 (PMC3276151; doi:10.1534/g3.111.000661)
Supplement: Supporting Information [file supp_1.5.343_FigureS4.pdf]

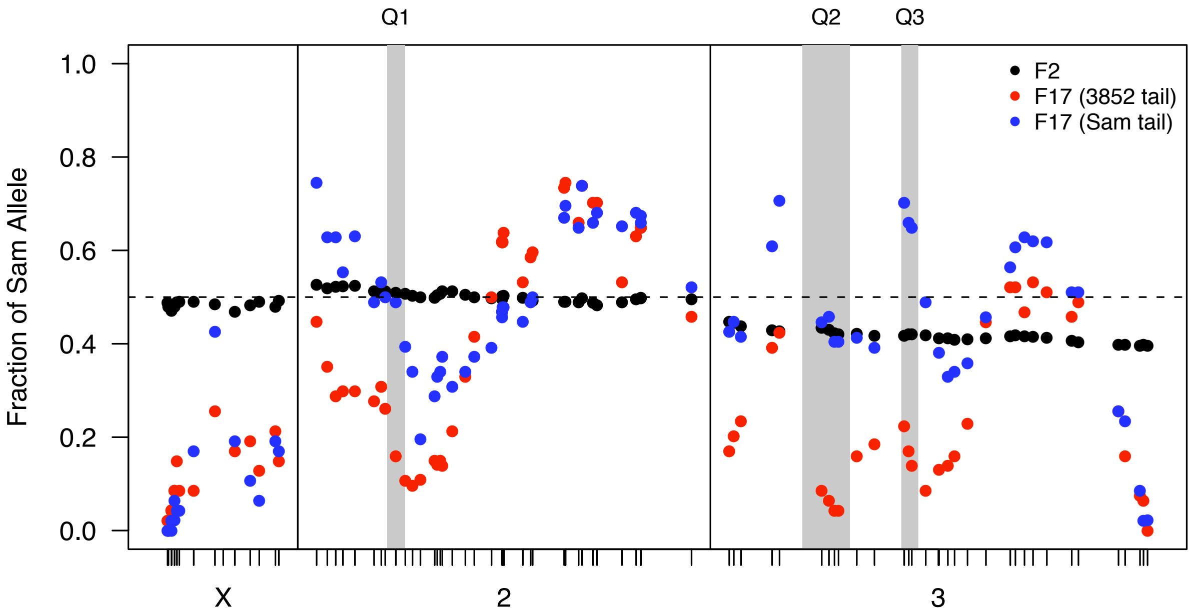

Figure S4 Frequency of the *Sam* allele at markers in the mapping panels. For every marker we directly count the fraction of alleles coming from the *Sam* parental line in each set of genotyped individuals - F_2 (black), b3852-like F_{17} tail (red), and *Sam*-like F_{17} tail (blue). The frequencies are plotted against the marker positions on the expanded F_{17} genetic map, and the three major QTL intervals are marked with gray boxes. In the F_2, *Sam* alleles are typically close to the expected frequency of 0.5, although frequencies are slightly lower for chromosome 3, potentially due to negative fitness consequences associated with the mutant ry^{506} allele. In the F_{17} both the X chromosome and the telomeric end of chromosome 3R show a dearth of *Sam* alleles in both tail samples, indicating mapping power in these regions is likely to be poor.
